# Supplementary material for: The role of mRNA-galsomes and LNPs in enhancing HIV-specific T cell responses across various lymphoid organs
Source: Mol Ther Nucleic Acids. 2024 Oct 28;35(4):102372. doi: 10.1016/j.omtn.2024.102372 (PMC11605416; doi:10.1016/j.omtn.2024.102372)
Supplement: Document S1. Figures S1–S5 and Table S1 [file mmc1.pdf]

OMTN, Volume *35*

Supplemental information

The role of mRNA-galsomes and LNPs in enhancing HIV-specific T cell responses across various lymphoid organs

Sigrid D'haese, Sabine den Roover, Rein Verbeke, Ilke Aernout, Sofie Meulewater, Joëlle Cosyns, Jessy Meert, Sarah Vanbellingen, Thessa Laeremans, Ine Lentacker, and Joeri L. Aerts

Supplemental material

Table S1: Antibodies used for flow cytometry analysis

Target	Fluorophore	Clone	Supplier
CD11c	PE-Cy-7a	N418	Biolegend
CD103	AF-700	2E7	
MHC-IS	APC	25-D1.16	
CD86	PE CF594	GL-1	
CD45.2	BV421	104	
	Spark NIR TM 685	104	
CD45.1	PE	A20	BD
CD3e	FITC	145-2C11	
CD4	PerCP-Cy5.5	RM4-5	
CD11b	PerCP-Cy5.5	M1/70	
CD8a	BV510	53-6.7	
IFN-γ	AF-700	XMG1.2	
TNF-α	PeCy7	MP6-XT22	
IL-2	APC	JES6-5H4	
MHC-II	PE	NIM-R4	eBioscience

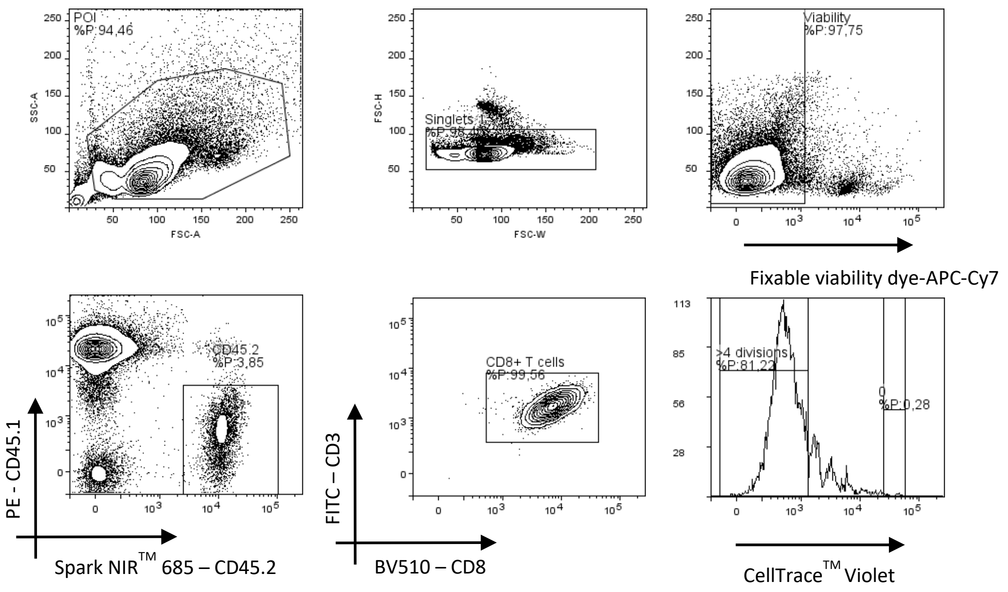

Figure S1: Gating strategy for the in vivo adoptive transfer assay. Viable cells were selected based on fixable viability dye and subsequently gated on CD45.2$^+$ to exclude host lymphocytes. Next, the transferred CD8$^+$ T cells were gated based on CD3 and CD8. Finally, proliferated cells were selected on dilution peaks, the final peak being the cells that divided more than four times.

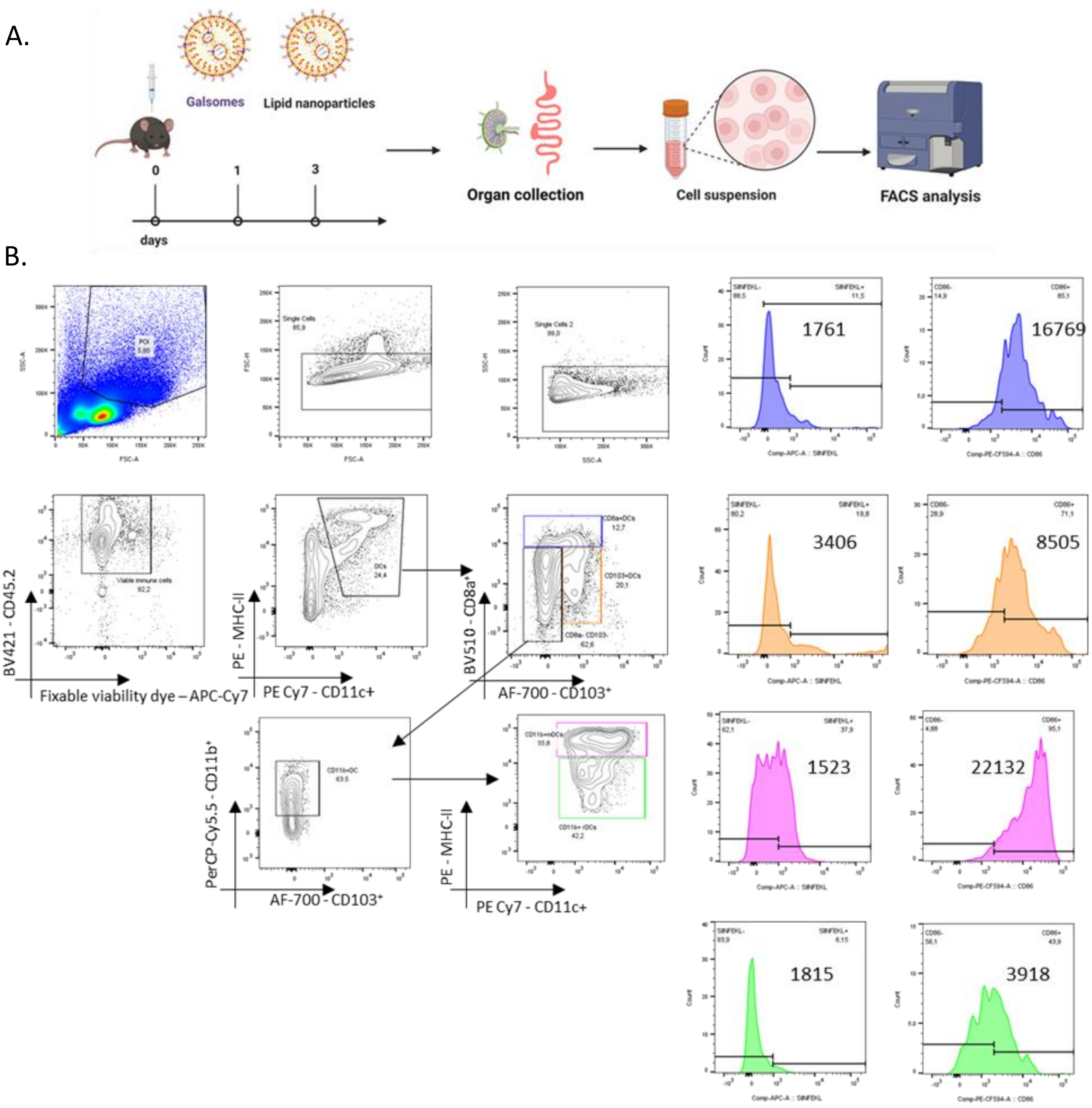

Figure S2. Experimental setup (A) and gating strategy antigen presentation and maturation in DCs in the ipsilateral lymph nodes (B). Gating was performed on the viable CD45.2$^+$ cells. DCs were gated based on MHC II and CD11c double positivity. Subsequently, CD8a$^+$ rDCs and CD103$^+$ mDCs were identified and based on the double negative population, the CD11b$^+$ m/r DCs were characterized. Within these subsets, presentation of SIINFEKL in MHC I and CD86 was assessed.

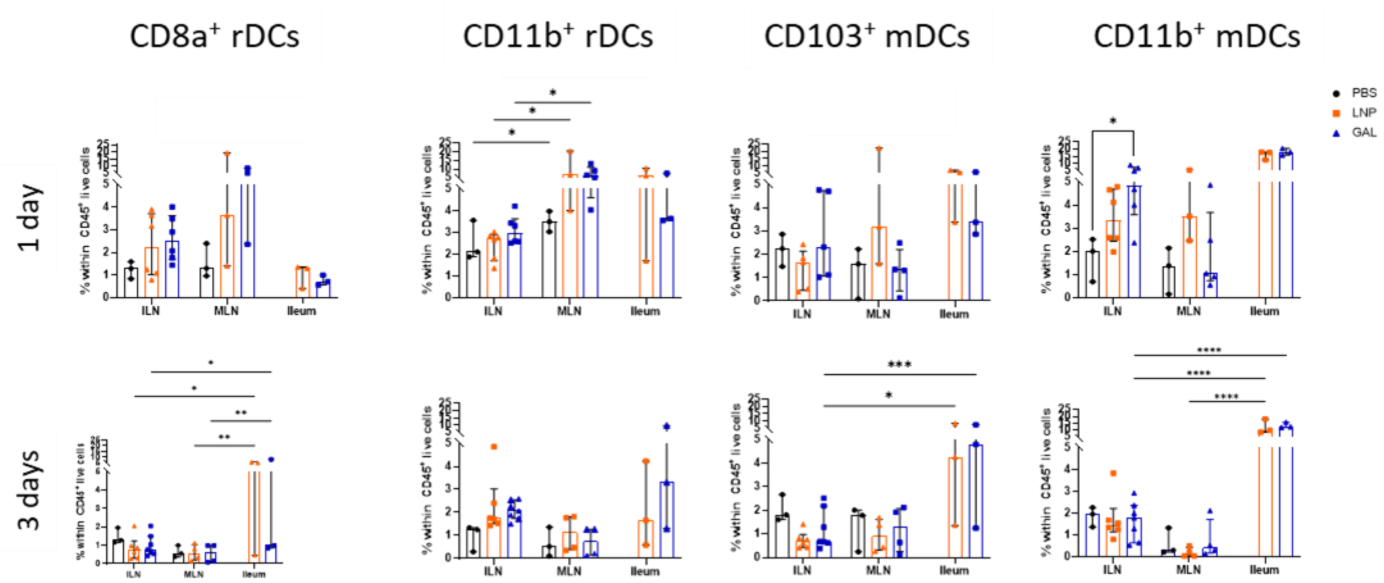

Figure S3. The identification of distinct DC subsets upon immunization with mRNA-LNP and mRNA-galsomes. The dynamics of various DC subsets in the inguinal (ILN) and mesenteric lymph nodes (MLN) and ileum following immunization with 2 µg Ψ/m^{5}C modified li80tOVA mRNA-LNP (LNP) and mRNA-galsomes (GAL). For ILN and MLN: each dot represents data from one mouse. For ileum: each dot represents data pooled from 2 mice. For the 24 and 72h time points, n=3 for PBS, n=6 for galsomes and n= 6. LNP. Median ± IQR. Mann-Whitney U-test, * p <0.05, ** p<0.01.

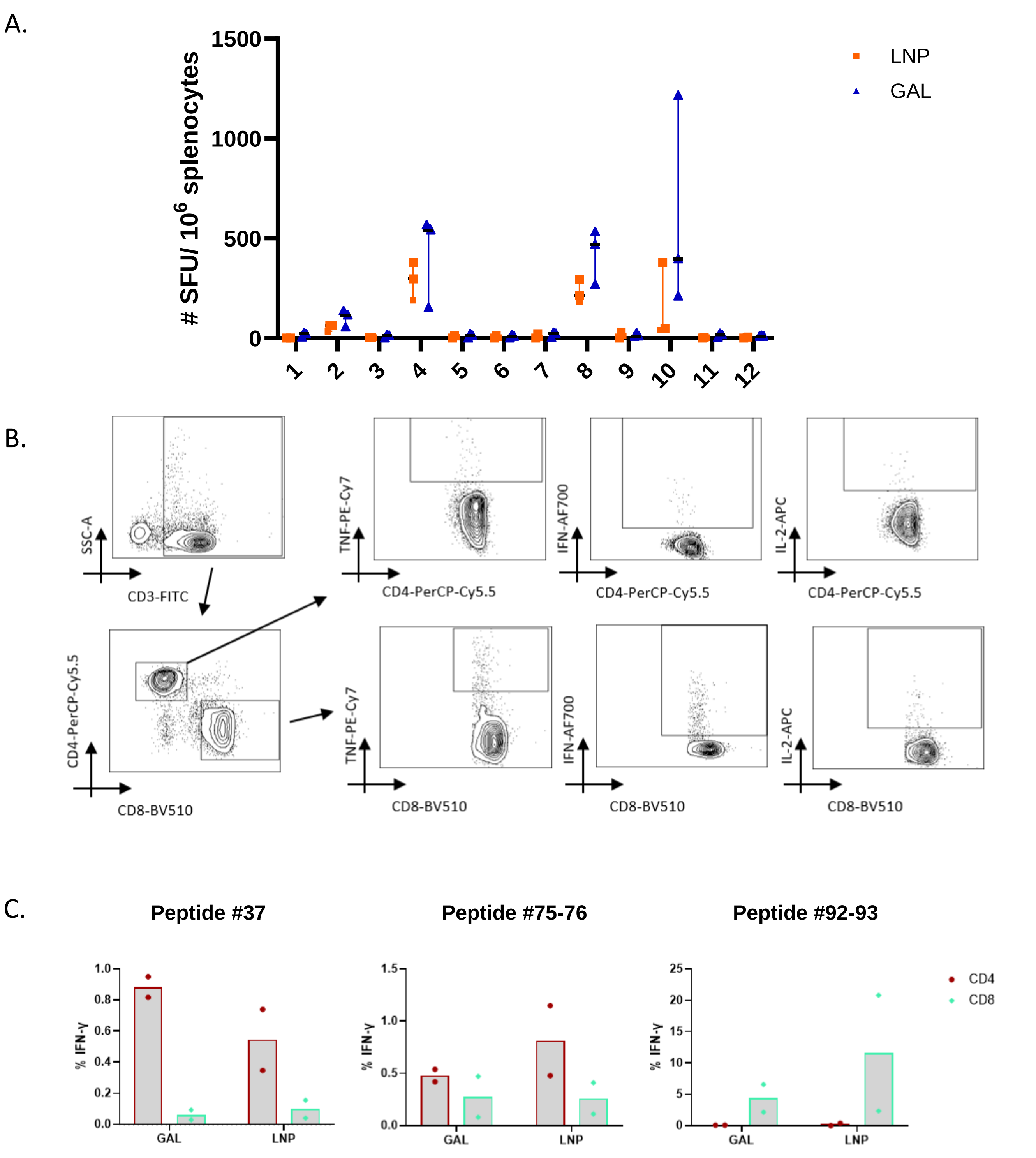

Figure S4. Identification of minimal $H2k^b$ stimulatory epitopes within HIV gag. A) ELISPOT for IFN-γ was performed for 12 separate gag-peptide pools each containing 10-12 overlapping peptides. B) Gating strategy intracellular cytokine staining. Splenocytes were gated based on CD3 and subsequently CD4 and CD8. Next, we looked at TNF, IFN and IL-2 production within the CD4 and CD8 population. C) IFN-γ secretion was assessed in splenocytes from immunized mice in $CD4^+$ and $CD8^+$ T cell separately. In brief, cells were thawed and peptides were added before overnight stimulation in the presence of monensin and brefeldin. n=2, each dot represents data from one mouse, bar represents mean.

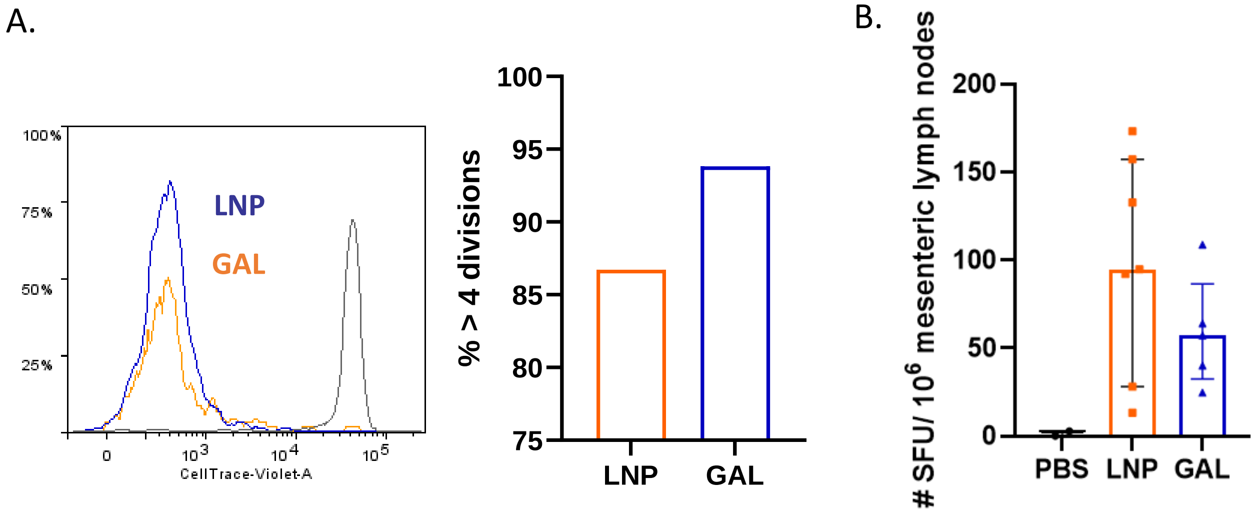

Figure S5. Immune response in the mesenteric lymph nodes. (A) $CD8^+$ T cells from OT-I mice were transferred to naive $CD45.1^+$ mice. After 2 days, naive $CD45.1^+$ mice were immunized with lipid nanoparticles (LNP) or galsomes (GAL) containing 2 µg ψ/m^5C-modified mRNA encoding li80tOVA. After 3 days, mice were sacrificed and proliferation of transferred $CD8^+$ T cells was assessed by flow cytometry in the mesenteric lymph nodes for one mouse in the GAL (blue) and LNP (orange) group. The PBS control is shown for the inguinal lymph nodes (gray). (B) Prime-boost immunization was performed with 2 µg Ψ/m^5C Gag mRNA containing galsomes and LNPs. Five days after boost injection, mesenteric lymph nodes were collected and used for IFN-γ ELISPOT analysis. Each symbol represents one mouse, n=5 for galsomes, n=7 for LNPs, n=2 for PBS controls. Median ± IQR, one outlier was removed in the GAL group.
