# Supplementary material for: The role of mRNA-galsomes and LNPs in enhancing HIV-specific T cell responses across various lymphoid organs
Source: Mol Ther Nucleic Acids. 2024 Oct 28;35(4):102372. doi: 10.1016/j.omtn.2024.102372 (PMC11605416; doi:10.1016/j.omtn.2024.102372)
Supplement: Document S2. Article plus supplemental information [file mmc2.pdf]

Molecular Therapy
Nucleic Acids
Original Article

The role of mRNA-galsomes and LNPs in enhancing HIV-specific T cell responses across various lymphoid organs

Sigrid D'haese,[1,5] Sabine den Roover,[1,5] Rein Verbeke,[2] Ilke Aernout,[2] Sofie Meulewater,[2] Joëlle Cosyns,[1,3] Jessy Meert,[4] Sarah Vanbellingen,[1] Thessa Laeremans,[1] Ine Lentacker,[2] and Joeri L. Aerts[1]

[1]Neuro-Aging and Viro-Immunotherapy (NAVI) Research Group, Faculty of Pharmacy and Medicine, Vrije Universiteit Brussel, Brussels, Belgium; [2]Ghent Research Group on Nanomedicines, Faculty of Pharmacy, Ghent University, Ghent, Belgium; [3]Hematology and Immunology (HEIM), Faculty of Pharmacy and Medicine, Vrije Universiteit Brussel, Brussels, Belgium; [4]Experimental Pharmacology (EFAR), Faculty of Pharmacy and Medicine, Vrije Universiteit Brussel, Brussels, Belgium

mRNA nanoparticles have been investigated in the context of prophylactic vaccination against HIV, but their effectivity has not been widely investigated in therapeutic vaccination. It has been suggested that a profound CD8$^+$ T cell response within lymphoid tissues, a primary site for viral reservoirs, is crucial for achieving optimal viral control, potentially correlating with protection. This study aimed to evaluate the effectiveness of mRNA lipid nanoparticles (LNPs), including a modified variant containing α-galactosylceramide as an adjuvant, termed galsomes. C57BL/6 mice were immunized intramuscularly with nucleoside-modified mRNA encoding ovalbumin (li80tOVA), revealing that mRNA-galsomes induced slightly higher proliferation levels of li80tOVA-specific CD8$^+$ T cells in lymphoid tissues across various anatomical sites compared with mRNA-LNPs. In addition, immunization with nucleoside-modified HIV-1 Gag mRNA elicited a notable Gag-specific immune response in both formulations even at low mRNA doses. Remarkably, mRNA-galsomes induced lower polyfunctional responses in CD4$^+$ T cells, but similar polyfunctional responses in CD8$^+$ T cells in the spleen compared with mRNA-LNPs. Importantly, the Gag-specific CD8$^+$ T cells demonstrated cytolytic capacity against target cells in both the spleen and lymphoid tissues, including gut-associated lymph nodes. These findings underscore the potential of both mRNA-galsomes and mRNA-LNPs as tools for therapeutic vaccination against HIV.

INTRODUCTION

Antiretroviral therapy (ART) has proven highly effective in improving the overall health and longevity of people living with HIV-1 (PLWH). Despite its success, the persistence of HIV-1 latent reservoirs in various anatomical sites including gut-associated and other lymphoid tissues presents a significant obstacle to viral eradication. These sites remain largely inaccessible to ART, contributing to viral persistence even during treatment.[1] In addition, discontinuation of ART typically leads to a rapid viral rebound in PLWH. As a result, long-term treatment is required, heightening the risk of developing comorbidities.[2–4] Therefore, we and others have explored therapeutic vaccines as a strategy to reinvigorate the host immune system, empowering it to control viral replication without the need for ART with the ultimate goal of achieving a functional cure.[5,6] In these studies, elite controllers, a select subset of PLWH, serve as a model for a functional cure due to their natural capacity to suppress viremia, maintain undetectable viral loads (<50 mL/copy), and elevate CD4$^+$ T cell counts without ART.[7–9] The primary drivers of this phenomenon are circulating viral suppressive CD8$^+$ T cells, which exhibit a high proliferative capacity and high levels of polyfunctionality. This is characterized by the expression of CD107a, interferon gamma (IFN-γ), macrophage inflammatory protein-1β, interleukin-2 (IL-2), tumor necrosis factor alpha (TNF-α), granzyme B, and perforin, which underscores their multifaceted antiviral activity.[7,10–13] Moreover, tissue-resident CD8$^+$ T cells play a pivotal role in suppressing viral replication in re-emerging viral reservoirs in the gut mucosa and other lymphoid tissues.[14] In addition to the CD8$^+$ T cells, innate invariant natural killer T (iNKT) cells have emerged as key players demonstrating heightened levels of polyfunctionality in HIV-1 controllers.[15] Conversely, iNKT cells demonstrated a faster depletion than CD4$^+$ T cells, this is demonstrated by the fact that, in PLWH, iNKT cells were decreased in both the periphery, where they support T cell responses, and the gut where they exert an anti-inflammatory function.[16–19] Furthermore, while ART treatment partially restores iNKT cell levels at both sites,[20,21] peripheral iNKT cells still show reduced proliferation and cytokine production.[15,22] An immunotherapy that strengthens both iNKT and CD8$^+$ T cell responses could therefore be a good vaccine candidate. MRNA-lipid nanoparticle (LNP)-based vaccines have showcased their ability to induce polyfunctional CD4$^+$ and CD8$^+$ T cell responses in the spleen of

Received 3 May 2024; accepted 24 October 2024;
https://doi.org/10.1016/j.omtn.2024.102372.

[5]These authors contributed equally

Correspondence: Joeri L. Aerts, NAVI, Vrije Universiteit Brussel, Laarbeeklaan 103, 1090 Brussels, Belgium.
E-mail: joeri.aerts@vub.be

Published by Elsevier Inc. on behalf of The American Society of Gene and Cell Therapy.
This is an open access article under the CC BY license (http://creativecommons.org/licenses/by/4.0/).

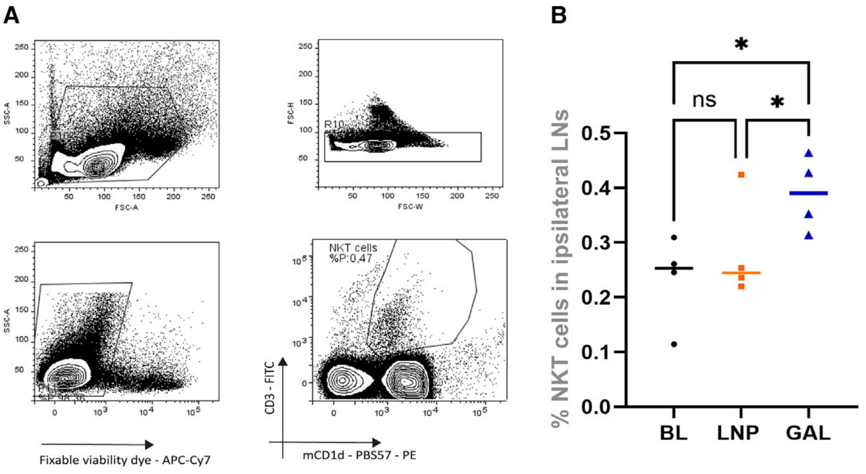

Figure 1. INKT cell proliferation in the draining lymph nodes

$CD45.1^+$ mice received an adoptive transfer of $CD45.2^+$ OT-I $CD8^+$ T cells. After 2 days these mice were immunized with LNPs or galsomes containing 2 μg ψ/m^5C-modified mRNA encoding li80tOVA. (A) Gating strategy for NKT cells. (B) Percentage NKT cells in ipsilateral lymph nodes. n = 4 for each group, baseline (BL) are unimmunized mice. Median ± interquartile range (IQR), Kruskal-Wallis test with multiple comparisons, *$p < 0.05$.

mice and human peripheral blood mononuclear cells (PBMCs).[23,24] Moreover, amid the COVID-19 pandemic, the mRNA-LNP vaccines, BNT162b2 and mRNA-1273, have exhibited excellent protection against symptomatic COVID-19 disease.[25,26] Recent clinical trials have demonstrated the efficacy of mRNA-LNP vaccines.[27,28] In contrast while prophylactic mRNA HIV vaccines are advancing (NCT05001371, NCT05414786, NCT05217641), therapeutic HIV vaccine research lags behind. In addition, while prophylactic immune responses are primarily focused on the development of HIV-envelope-specific neutralizing antibodies, therapeutic vaccination necessitates robust Gag-specific polyfunctional $CD8^+$ T cell responses.[29–32] Despite their crucial role, gut lymphoid tissues are often excluded in both analysis and preclinical studies. So far, only a handful of preclinical studies in mice or macaques have explored mRNA-LNP formulations for inducing Gag-specific polyfunctional $CD8^+$ T cell responses.[33–35] Notably, a recent study demonstrated that the glycolipid adjuvant α-galactosylceramide (α-GC) (mRNA-galsomes) leads to the co-activation of conventional T and iNKT cells.[36] The activation of iNKT cells by galsomes, which bind α-GC presented in CD1d molecules on antigen-presenting cells (APCs) initiates a cascade of events. Subsequently, these activated iNKT cells initiate the CD40-CD40L crosstalk with dendritic cells (DCs) leading to the secretion of IL-12 secretion and the upregulation of the maturation markers CD80 and CD86. This process prompts the release of elevated levels of IFN-γ, TNF-α, IL-2, and IL-4. This in turn provides stimuli for the activation of B and T cells.[36–38] Therefore, we hypothesize that galsomes might provide an efficient therapeutic HIV-1 vaccine candidate by restoring iNKT functionality and inducing $CD8^+$ T cell-mediated responses. In this paper, we evaluated an LNP platform comprising the C12:200 ionizable lipid and nucleoside-modified mRNA encoding the Gag consensus HxB2 model antigen in C57BL/6 mice and compared it with the same vaccine formulation including α-GC, mRNA-galsomes.[36] Initially, we evaluated the proliferative capacity of antigen-specific $CD8^+$ T cells following intramuscular immunization with both vaccines. In addition, we analyzed antigen presentation in the context of MHC class I and maturation in various DC subsets, comparing the inflammatory potential of both. Subsequently, we investigated the Gag-specific immune responses to different doses of mRNA-LNPs or mRNA-galsomes. Finally, we assessed polyfunctional $CD4^+$ and $CD8^+$ T cell responses in the spleen as well as the cytotoxic capacity in the spleen, and inguinal and mesenteric lymph nodes.

RESULTS

mRNA-galsomes activate iNKT cells and induce strong antigen-specific proliferation of $CD8^+$ T cells

The design of the galsomes was tailored to allow APCs to interact with conventional T cells and iNKT cells during the immunization process. We first evaluated the capacity of mRNA-galsomes to expand iNKT cells in the draining lymph nodes, drawing upon existing literature[36] and recent data by Meulewaeter et al.[39] As anticipated, mice immunized with mRNA-galsomes exhibited a higher percentage of iNKT cells (gating strategy in Figure 1A) in the inguinal lymph nodes draining in the injection site (ipsilateral) lymph nodes compared with baseline and mice immunized with mRNA-LNP, 3 days post-immunization (Figure 1B). To assess the expansion of antigen-specific $CD8^+$ T cells in various lymphoid tissues, we employed the model antigen li80tOVA (a truncated form of ovalbumin fused with the first 80 amino acids of the invariant chain [li80] and a non-secreted variant of ovalbumin [tOVA]) in an *in vivo* adoptive transfer assay using OT-I $CD8^+$ T cells. Two days after intravenous transfer of OT-I $CD8^+$ T cells ($CD45.2^+$), $CD45.1^+$ mice were immunized with mRNA-galsomes or mRNA-LNPs containing li80tOVA encoding nucleoside-modified mRNA (5-methylcytidine [m^5C] and pseudouridine [ψ]). To track the OT-I cells *in vivo*, we employed the mismatch between CD45.1 and CD45.2 (gating strategy in Figure S1). The percentage of OT-I $CD8^+$ T cells undergoing more than four divisions was assessed in the spleen and the ipsilateral or contralateral (draining lymph nodes on the opposite injection side) lymph nodes (Figure 2A). Nearly 100% of the transferred $CD8^+$ OT-I T cells underwent four divisions in both groups in the ipsilateral lymph nodes (Figure 2B). However, upon mRNA-galsomes immunization, the percentage of $CD8^+$ OT-I T cells that underwent more than four divisions was 5%–10% higher in the spleen and contralateral lymph nodes, respectively, compared with mice immunized with mRNA-LNPs.

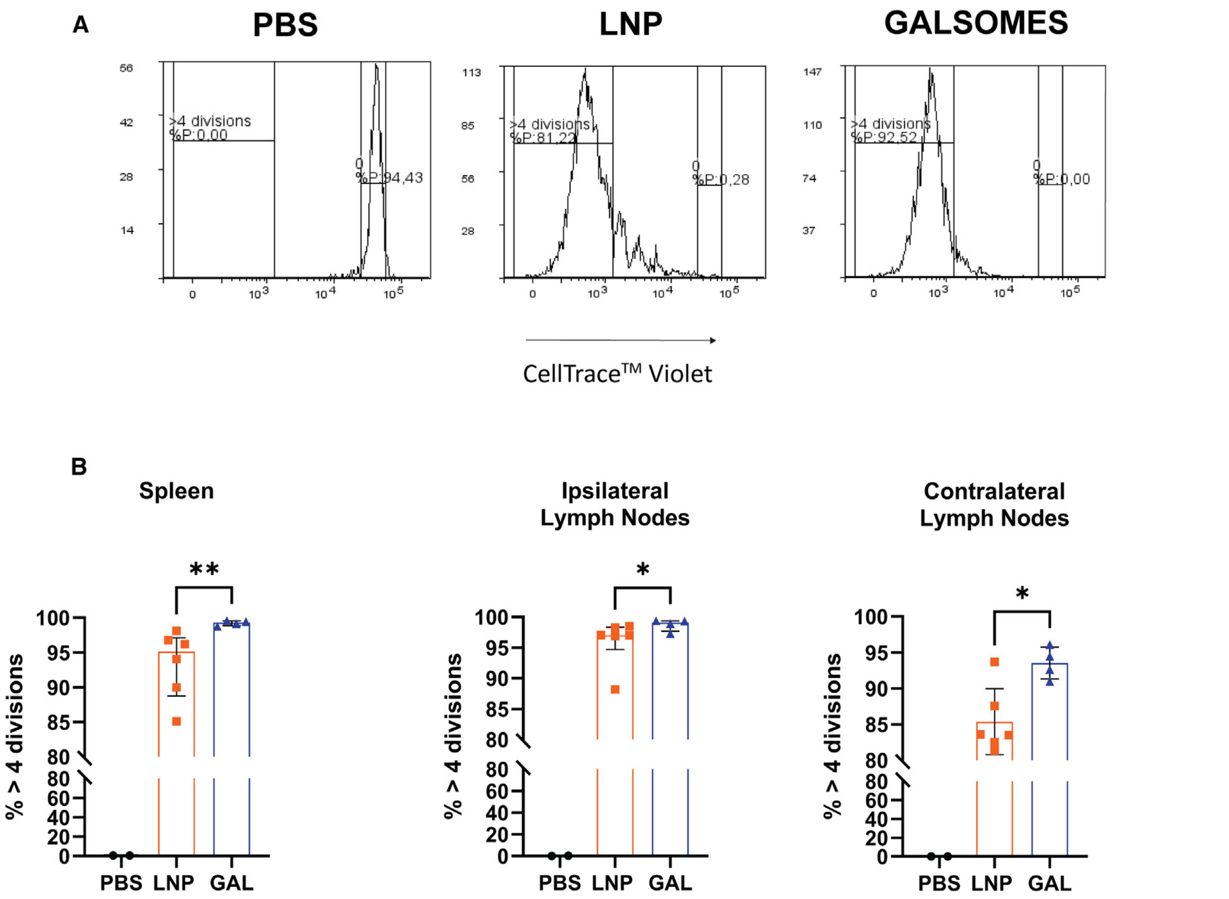

Figure 2. Proliferation of antigen-specific CD8$^+$ T cells upon immunization with mRNA-LNPs and galsomes

CD8$^+$ T cells obtained from OT-I mice were labeled with CellTrace Violet (CTV) and subsequently transferred into naive CD45.1$^+$ mice. Two days post-transfer, the CD45.1$^+$ mice were immunized intramuscularly with LNPs or galsomes (GAL) containing 2 μg of ψ/m^{5}C-modified mRNA encoding li80tOVA. Three days post-immunization, the mice were sacrificed and the proliferation of transferred CD8$^+$ T cells was assessed via flow cytometry in both the spleen and inguinal lymph nodes. (A) Example of the gating strategy identifying the population that had undergone more than four divisions. (B) The percentage of OT-I cells that divided more than four times was quantified for both the spleen and inguinal lymph nodes on the ipsilateral (draining) and contralateral (non-draining) sides of the injections. Each symbol represents one mouse, GAL $n = 4$, LNP $n = 6$, PBS $n = 2$, median ± IQR, Mann-Whitney U test, *$p < 0.05$, **$p < 0.01$.

mRNA-galsomes give rise to antigen presentation, maturation, and inflammasome activation in DCs

Following this, we explored the DC subset responsible for presenting the highly immunogenic epitope SIINFEKL from the model antigen li80tOVA in MHC class I. Classical or conventional DCs (cDCs) are divided into two distinct functional lineages: the CD8a$^+$ (CD103$^+$) cDC1 lineage and the CD11b$^+$ cDC2 lineage, depending on their presence in various lymphoid tissues. Generally, cDC1 cells are known for cross-presenting extracellular antigens to CD8$^+$ T cells, while cDC2 cells have a lower capacity for cross-priming and are more involved in promoting CD4$^+$ T cell responses. To investigate which DC subset is responsible for presenting SIINFEKL in MHC class I, we isolated inguinal lymph nodes 1 and 3 days after intramuscular immunization with mRNA-galsomes and mRNA-LNPs containing 2.5 μg of modified mRNA li80tOVA. Given the vital role of the gut and its associated tissues as a viral reservoir,[1] we also profiled the DC subsets in the mesenteric lymph nodes and ileum. We examined the presence of resident (r) DCs (CD8a$^+$cDC1 and CD11b$^+$cDC2) and migratory (m) DCs (CD103$^+$ cDC1 and CD11b$^+$ cDC2) in these different lymphoid tissues (gating strategy outlined in Figure S2).

One day post-immunization an increase in the CD11b$^+$ mDC subset was observed in the inguinal lymph nodes after immunization with mRNA-galsomes (Figure 3A). In the mesenteric lymph nodes, it was primarily immunization with mRNA-LNPs that led to an increased presence of residential CD8a$^+$ and CD11b$^+$ DCs compared with other DC subsets. However, after 3 days (Figure S3) a decreasing trend was observed across all DC subsets. While this trend was also observed in the inguinal lymph nodes (Figure 3A), a statistically significant upregulation of the maturation marker CD86 was observed within the CD11b$^+$ mDCs compared with other DC subsets

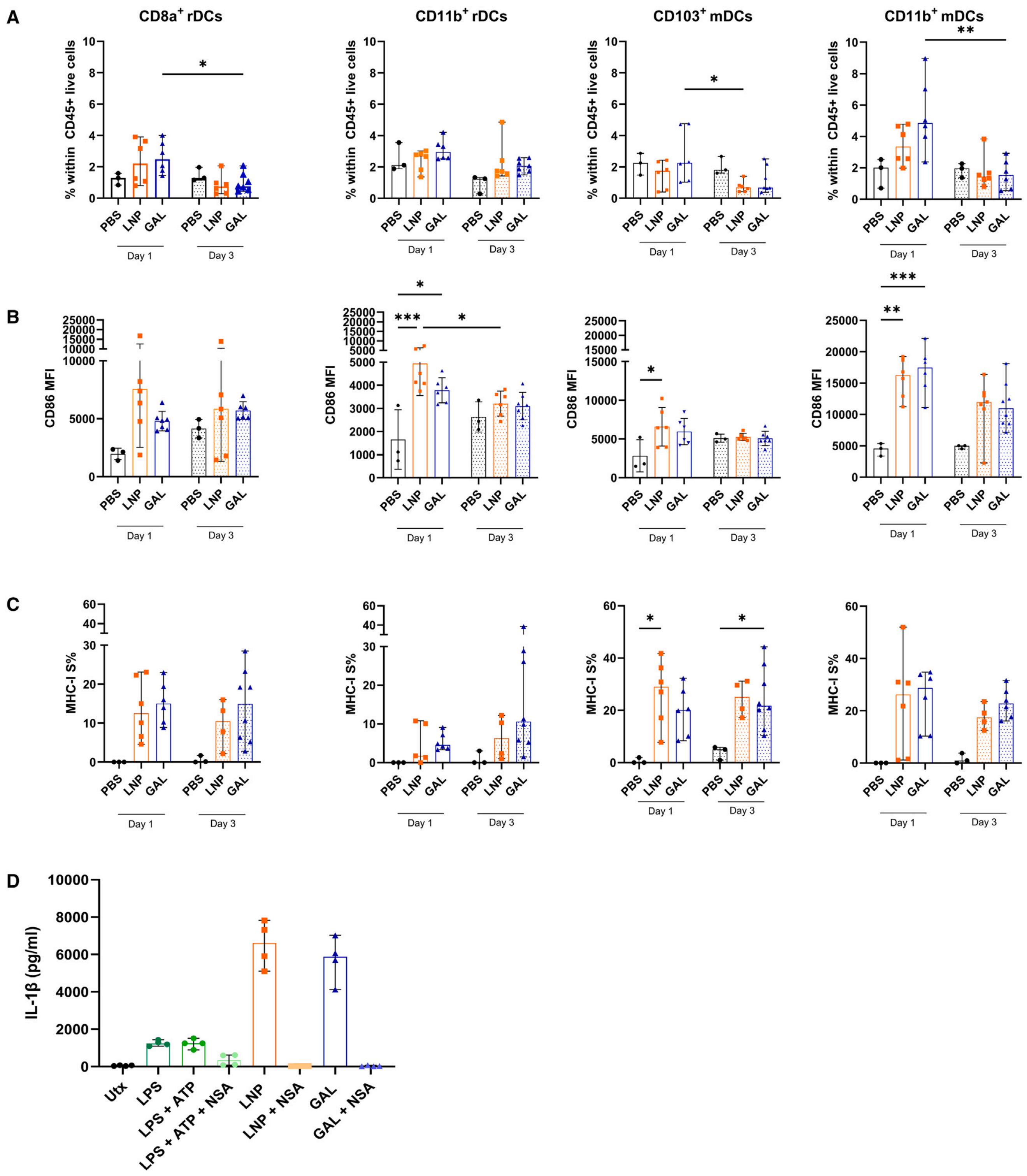

(legend on next page)

(Figure 3B). However, mRNA-galsomes induced a persistent and even an increased maturation level within the CD8a$^+$ rDCs subset over time. Despite the lack of an increased proportion of CD103$^+$ DCs in the inguinal lymph nodes post-immunization with both vaccines, this subset, alongside CD8a$^+$ rDCs and CD11b$^+$ mDCs, demonstrated effective antigen presentation of SIINFEKL in MHC class I. Overall, after 3 days, the levels of SIINFEKL presentation remained consistent across all DC subsets (Figure 3C).

Recent findings highlighted that mRNA-LNP formulations induce high levels of IL-1β in human PBMCs.[40] To explore whether this phenomenon holds true for our formulations, we generated bone marrow-derived DCs (BMDCs) and incubated the cells overnight with nucleoside-modified mRNA-galsomes or mRNA-LNPs. The BMDCs secreted substantial amounts of IL-1β and this response could be blocked by the inhibitor necrosulfonamide (NSA), an inhibitor of gasdermin D, which is essential for the release of IL-1β. This indicates that both nucleoside-modified mRNA-LNPs and mRNA-galsomes possess inflammatory potential at least in an *ex vivo* context (Figure 3D).

Immunization with mRNA-galsomes and mRNA-LNPs results in Gag-specific T cell responses displaying a similar antigen-breadth

In a first step, we sought to determine the optimal immunization dose for Gag consensus HxB2 mRNA. Mice were immunized with mRNA-galsomes or mRNA-LNPs containing 0.5, 2.5, or 5 μg of nucleoside-modified mRNA. An ELISPOT assay was employed to quantify IFN-γ secretion in splenocytes after stimulation with a Gag-peptide pool, conducted 5 days post-boost immunization. For both formulations, no differences were observed among the various mRNA doses (Figure 4A).

Following this, we consolidated data from two experiments involving distinct batch formulations of mRNA-LNPs and mRNA-galsomes encapsulating 2 μg of Gag-mRNA: one was used immediately after production, while the other was tested after frozen storage. No difference in IFN-γ secretion was observed between the two batches (Figure 4B), demonstrating that the formulations maintained functionality upon freezing.

mRNA-LNPs promote higher levels of polyfunctionality in CD4$^+$ T cells while similar effects were observed in CD8$^+$ T cells for both formulations

Given the significance of not only evaluating the response against the entire Gag antigen but also considering the number of recognized epitopes,[32] we assessed the breadth of the immune response by segregating the 113 overlapping Gag peptides into 12 peptide pools each consisting of 10–12 overlapping peptides. Notably, pools 4, 8, and 10 were identified as containing epitopes that induced immune responses (Figure S4A). Overall, these results indicated that mice immunized with either mRNA-LNPs or mRNA-galsomes elicited comparable immune responses.

By further separating out the reactive pools down to the individual peptide level, we identified five immune-stimulatory peptides, which were subsequently utilized to gauge the level of polyfunctionality. To achieve this, we performed intracellular cytokine staining (gating strategy in Figure S4B) following stimulation with these peptides individually. Interestingly, we observed that peptide 37 (QAISPRTLNAWVKV) predominantly stimulated CD4$^+$ T cells, whereas peptide 75 (VDRFYKTLRAEQASQ) and 76 (YKTLRA EQASQEVKN) acted as mixed CD4$^+$ and CD8$^+$ T cell stimulators and peptide 92 (EAMSQVTINSATIMMQ) and 93 (QVTNSA TIMMQRGNF) exclusively stimulated CD8$^+$ T cells (Figure S4C). Subsequently, for a more comprehensive characterization of the cellular response, intracellular cytokine staining was performed to evaluate the production of IFN-γ, TNF-α, and IL-2 by the CD4$^+$ and CD8$^+$ T cell subsets. The percentage of CD4$^+$ T cells producing IFN-γ and IL-2 were nearly identical for both mRNA-LNPs and mRNA-galsomes (Figure 5A). In addition, a trend toward higher TNF-α production was observed in the CD4$^+$ T cell subset for mRNA-LNPs compared with mRNA-galsomes.

Regarding the CD8$^+$ T cells, the production of IL-2 was relatively low for both mRNA-LNP and mRNA-galsomes-immunized mice (Figure 5B). However, the production of TNF-α was equally high for both mRNA-LNP and mRNA-galsomes immunization. Lastly, there was a small trend toward increased production of IFN-γ in the CD8$^+$ T cell subset for mRNA-galsomes-immunized mice compared with mRNA-LNPs. When summarizing these data and considering polyfunctionality, a substantial proportion of CD4$^+$ T cells expressed more than one cytokine and the overall number of CD4$^+$ T cells producing any cytokine was significantly higher for mRNA-LNP-immunized mice (Figure 5C). Due to the low production of IL-2, we observed a low proportion of polyfunctional CD8$^+$ T cells. Nevertheless, the percentage of CD8$^+$ T cells producing at least one cytokine remained high for both formulations (Figure 5D). Overall, although the polyfunctional CD4$^+$ T cell response was lower for mRNA-galsomes-immunized mice, the polyfunctional CD8$^+$ T cell response exhibited similarity between both vaccines.

mRNA-LNPs and mRNA-galsomes induce a Gag-specific cytotoxic immune response in the spleen and lymphoid tissues

To assess the cytotoxic capability of the CD8$^+$ T cells *in vivo*, we conducted an *in vivo* cytotoxicity assay. In this assay, splenocytes are

Figure 3. Antigen presentation and maturation of DCs *in vivo* and inflammasome activation *ex vivo*

(A–C) At 1 and 3 days after immunization with either vehicle (PBS), LNPs or galsomes (GAL) containing 2 μg ψ/m^{5}C-modified mRNA encoding Ii80tOVA, the ipsilateral lymph node was harvested. After homogenization, cells were stained for DC markers. Within each DC subset (A), the MFI of CD86 (B), and the percentage presentation of SIINFEKL within the context of MHC class I (C) were assessed. Each symbol represents one mouse, $n = 3$ for PBS, $n = 6$ for mRNA-galsomes, and $n = 6$ for mRNA-LNP, median ± IQR, Mann-Whitney U test, *$p < 0.05$, **$p < 0.01$. (D) Bone marrow-derived DCs (BMDCs) were stimulated for 17 h with LPS with or without ATP or with LNP or GAL containing 2 μg of ψ/m^{5}C-modified mRNA encoding Gag HxB2 with or without the presence of NSA. $n = 4$, mean ± SD.

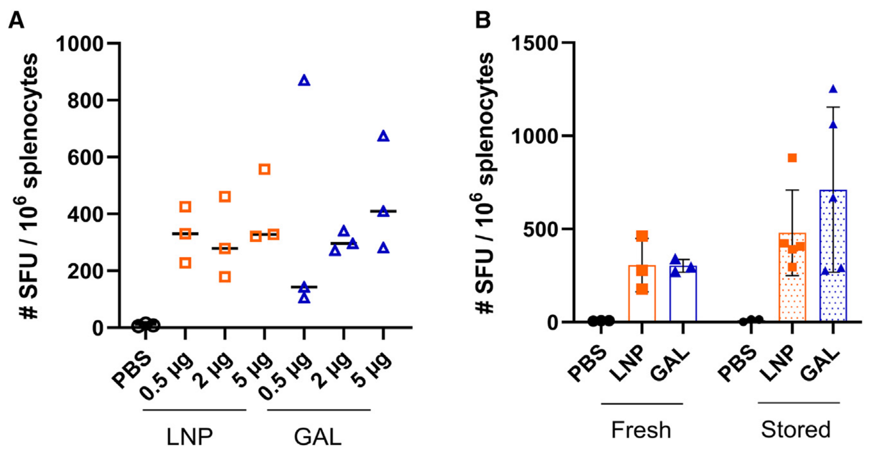

Figure 4. Dose-response rate and antigen breadth after immunization with nucleoside-modified Gag mRNA-LNPs and galsomes

C57BL/6 mice were immunized with 0.5, 2, or 5 μg ψ/m^{5}C-modified Gag HxB2 mRNA encapsulated in LNP (orange) or galsomes (GAL) (blue) following a prime-boost regimen with a 2-week interval. Five days after boost immunization, spleens were collected and splenocytes were analyzed via ELISPOT for IFN-γ secretion after stimulation with a Gag-peptide pool consisting of 113 overlapping peptides. (A) For dose determination, mice were immunized with 3 different doses, as indicated on the graph. (B) Summary of the mice receiving either the initial mRNA-LNP or mRNA-galsomes formulation (fresh) or the frozen formulation (stored), summary of 2 experiments, with n = 8 for immunized mice, n = 6 for PBS controls. Median values are displayed on the graph, Mann-Whitney U test, p value shown on graph.

pulsed with the aforementioned five selected Gag peptides and we assessed antigen-specific lysis of target cells in the spleen as well as in the lymph nodes (Figure 6A). Considering the significant role of the gut and the gut-associated tissues as a viral reservoir, which persists despite ART,[1] and taking into account the higher total levels of CD8$^+$ T cells and a greater proportion of these cells exhibiting polyfunctional characteristics observed in elite controllers compared with PLWH on ART,[41,42] we expanded our investigation beyond the spleen and inguinal lymph nodes. Specifically, we examined the mesenteric lymph nodes, which are crucial sites for instructing priming and differentiation of adaptive immune cells at the gut level.[43]

Across all immunized mice, we observed antigen-specific lysis in both the spleen and lymph nodes. Notably, there was no discernible difference between mice immunized with mRNA-galsomes or -LNPs; all mice demonstrated antigen-specific cytotoxicity, albeit with great variability (Figure 6B).

Moreover, we also observed the proliferation of OT-I CD8$^+$ T cells (Figure S5A) and detected IFN-γ secretion via ELISPOT after prime-boost immunization with Gag-mRNA LNPs and mRNA-galsomes (Figure S5B). In summary, the cytotoxic immune response for mRNA-galsomes- and mRNA-LNP-immunized mice was comparable and even extended to secondary lymphoid tissues.

DISCUSSION

In this paper, we demonstrate that immunization with nucleoside-modified mRNA, whether formulated in LNPs with or without α-GC, robustly induces a cellular immune response. For the development of a therapeutic HIV vaccine, it is crucial that this cellular immune response demonstrates proliferation, polyfunctionality, and cytotoxicity. In addition, the presence of CD8$^+$ T cells is essential not only in the periphery but also and especially within lymphoid tissues and the gut.[11,41,44,45] The cellular immune response induced by mRNA-LNPs or mRNA-galsomes, as described in this paper, fulfils these requirements.

mRNA-galsomes were designed to enhance iNKT cell responses, as demonstrated by an increase in the percentage of iNKT cells in the draining lymph nodes 3 days post-injection in C57BL/6 mice. We initially assessed the ability of nucleoside-modified mRNA-LNPs and mRNA-galsomes to induce a proliferative antigen-specific immune response using the model antigen ovalbumin. We observed high levels of proliferation of li80tOVA-specific CD8$^+$ T cells across all analyzed lymphoid tissues. Notably, the mRNA-galsomes formulation elicited slightly higher proliferation in lymphoid tissues distant from the draining lymph node, which we attribute two potential factors.

Firstly, this could be due to the migration of APCs to more distant tissues, where they could engage in subsequent antigen presentation. A similar phenomenon was observed with the COVID-19 vaccine candidate CvnCoV in macaques, where higher doses led to greater dissemination of mRNA-containing APCs to tertiary draining lymph nodes, potentially enhancing the initiation of adaptive immunity.[46] However, in the limited number of samples where sufficient material was available for analysis, we did not detect antigen presentation in the spleen, and contralateral or mesenteric lymph nodes, making this explanation less likely. Secondly, if DC migration is not responsible, it is possible the proliferating CD8$^+$ T cells exhibit altered migration patterns. For instance, it has been reported, that iNKT cells can recruit CCR4$^+$ CD8$^+$ T cells to lymph nodes, which may subsequently home to peripheral tissues in mice.[47,48] In the context of HIV, CXCR5 is a relevant homing marker, as CXCR5$^+$ CD8$^+$ T cells entering the B cell follicles have been associated with viral control in PLWH.[41]

Drawing from the observation that *Batf3*, a gene instructing CD103$^+$ mDC development played a crucial role in eliciting a CD8$^+$ T cell response after vaccination with BNT162b2,[49,50] we sought to identify the specific DC subset responsible for presenting li80tOVA peptide SIINFEKL in MHC class I. Unexpectedly, not only were CD103$^+$ mDCs responsible for antigen presentation to CD8$^+$ T cells, but

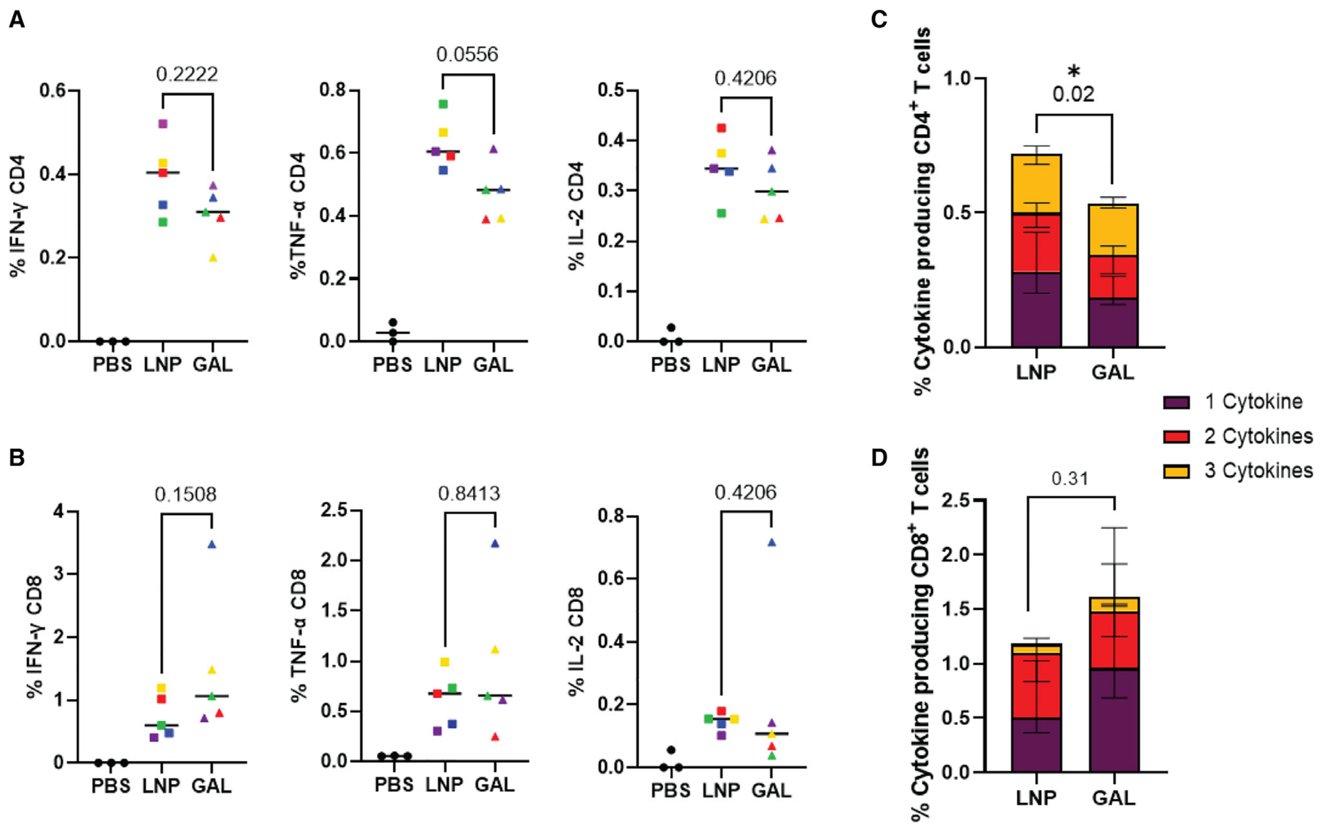

Figure 5. T cell polyfunctionality in the spleen of immunized mice

C57BL/6 mice received a prime-boost immunization with 2 μg ψ/m^{5}C-modified Gag mRNA formulated by LNPs or galsomes (GAL). Five days post-immunization, splenocytes were collected and placed in co-culture with DC2.4 cells pulsed with five selected Gag-peptides. The following day, intracellular cytokine staining was performed to analyze the simultaneous production of IFN-γ, TNF-α, and IL-2 within CD4$^+$ and CD8$^+$ T cells. CD4$^+$ (A) and CD8$^+$ (B) T cells producing each of the cytokines. Each symbol represents an individual mouse (color coded), with $n = 3$ for the PBS group and $n = 5$ for immunized groups, Mann-Whitney U test, p value displayed on graph. Boolean gating was applied to analyze the co-expression of the aforementioned cytokines for CD4$^+$ T cell (C) and CD8$^+$ T cell (D) subsets. Median ± IQR per subdivision, Mann-Whitney U test performed on the sum of 3 functions, p value displayed on graph, $^*p < 0.05$.

also CD8a$^+$ rDCs and CD11b$^+$ mDCs. Following immunization with mRNA-galsomes and mRNA-LNPs, we observed a significantly higher expression of CD86 on the CD11b$^+$ mDCs in the inguinal lymph nodes compared with the other DC subsets, which declined by day 3. While CD103$^+$ mDCs (cDC1) are primarily responsible for antigen cross-presentation during LNP immunization,[49,50] our data demonstrate that their migratory CD11b$^+$ cDC2 counterparts are also proficient in inducing effective SIINFEKL presentation in MHC class I and displaying elevated maturation levels. Previous studies suggested that CD11b$^+$ mDCs can engage in cross-presentation, under certain conditions, but further investigation is needed to validate this hypothesis.[51,52] Furthermore, our study exclusively focused on myeloid DC subsets and other APCs such as plasmacytoid DCs, macrophages, and monocytes were not taken into consideration. Nevertheless, mRNA-galsomes increased CD86 levels in the CD8a$^+$ rDC subset for up to 3 days. In theory, this could be due to the crosstalk between iNKT cells and DCs, potentially explaining the stronger proliferation in distant tissues. However, further investigation is needed.[37,53]

Recent studies have shown that mRNA-LNPs containing ionizable lipids are inflammatory and promote assembly of the nod-like receptor family pyrin domain containing 3 (NLRP3). This is likely due to reactive oxygen species generated from endosomal disruption, which elevates IL-1β levels in human PBMCs.[40,54] This inflammatory response is triggered by mRNA activating melanoma differentiation-associated protein 5 (MDA5) and retinoid acid-inducible gene (RIG-I) leading to the production of components and pro-inflammatory cytokines necessary for inflammasome formation.[49] The inflammatory properties of the ionizable lipids within the cell further contribute to activating this process. In line with these findings, we observed that both nucleoside-modified mRNA-galsomes and mRNA-LNPs induced comparable levels of IL-1β secretion, which can be attributed to the inherent adjuvant activity of the mRNA-LNPs.

In the next phase, mRNA-LNPs and mRNA-galsomes were evaluated using the model HIV antigen Gag HxB2. Even at a low dose of 0.5 μg mRNA, which is considerably lower than the typical

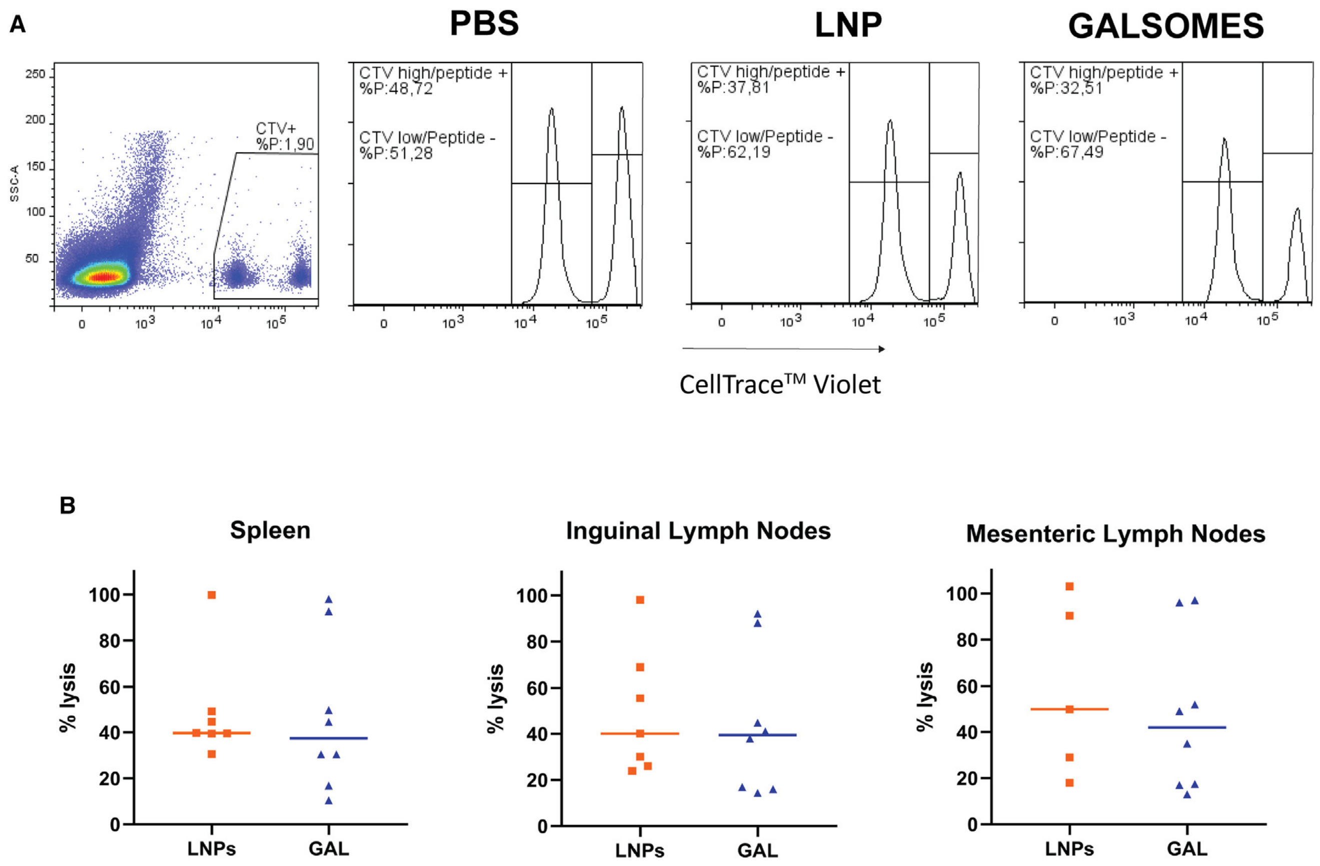

Figure 6. Cytotoxic immune response in spleen and lymphoid tissues of immunized mice

C57BL/6 mice were immunized in a prime-boost regimen with 2 μg ψ/m^{5}C-modified mRNA encoding Gag formulated either in LNPs or galsomes (GAL). Four days post-immunization, a pool of splenocytes was prepared, with one subset labeled with a high concentration of CTV and pulsed with the five selected Gag peptides, while another subset was labeled with a low concentration of CTV and left unpulsed. These labeled cells were then injected in the immunized mice. The following day, the spleen, and mesenteric and inguinal lymph nodes were collected and percentage lysis was calculated based on the PBS controls and the percentage reduction in the CTV high peak. (A) Example of flow cytometry data. (B) Percentage lysis in spleen and lymph nodes, with each symbol representing one mouse, n = 8 (7 for inguinal lymph nodes) for galsomes, n = 7 (5 for mesenteric lymph nodes) for LNPs median is represented on graph.

optimal doses (3–37.5 μg mRNA) used in most mouse preclinical studies, antigen-specific secretion of IFN-γ was observed.[33,34,55,56] Although there was an impact on antigen-specific proliferation, both formulations showed similar levels of IFN-γ secretion after a prime-boost immunization. It is possible that the second dose resulted in a maximal immune response for both formulations, hindering the ability to demonstrate any differences. In addition, both formulations elicited an equally broad immune response, with the same 5 peptides out of a total of 113 standing out in the re-stimulation of T cell responses. The identified primary epitope-containing peptides, could not be traced back to the Los Alamos database,[57] possibly due to the predominant description of H-2K^d epitopes suggesting a potential underreporting of studies utilizing C57BL/6 mice. We did observe a mild stimulatory effect of the SLYNTVATL-containing peptide, the most immunogenic epitope for the HLA-A2 allele in humans, which has also been described for C57BL/6 mice.[58] However, it performed less effectively than the other defined peptides and was consequently excluded from subsequent experiments.

The identification of the five immunogenic Gag peptides played a crucial role in subsequent functional investigations, as both CD4$^+$ and CD8$^+$ T cell epitopes were discerned. Polyfunctionality is a major determinant of the quality of HIV-specific T cell responses. In addition, recent recommendations for HIV therapeutic vaccines highlighted the importance of minimizing CD4$^+$ T cell stimulation while maximizing CD8$^+$ T cell response to avoid expanding or (re-)activating HIV-specific CD4$^+$ T cells, which are a major part of the viral reservoir.[58,59] Upon investigating cytokine levels, we found that mRNA-galsomes, despite “minimal” CD4$^+$ T cell responses, induced CD8$^+$ polyfunctional T cell responses similar to mRNA-LNP. Notably, both formulations produced CD8$^+$ T cell responses comparable with those seen with other HIV mRNA-based LNPs in similar animal models and generated a higher percentage of polyfunctional

CD4$^+$ and CD8$^+$ T cell responses compared with a PEI-based nanoparticle formulation.[33,34,60]

Subsequently, we assessed the cytotoxic potential of CD8$^+$ T cells directly *in vivo*, instead of using surrogate markers such as CD107a, granzyme B, and perforin. Our findings indicate a 100% response rate and a median of 40% antigen-specific lysis after two doses of only 2.5 μg mRNA. This surpasses the efficacy observed in other studies on mRNA-based therapeutic vaccination against HIV in mice, albeit with different antigens.[33,55]

Considering the emerging role of (gut-associated) lymphoid tissue in HIV persistence and the imperative for an efficient therapeutic vaccine to elicit a polyfunctional immune response at these sites, our search for vaccine formulations amenable to mucosal immunization has intensified. As this often involves oral vaccination, special care needs to be taken to protect the vaccine against the hostile gastrointestinal environment. To our knowledge, no mRNA formulation has been developed for oral immunization.[61] In addition, we are the first to investigate antigen-specific T cell responses in the mesenteric lymph nodes within a preclinical mRNA vaccination study, alongside more commonly studied lymphoid tissues. The strong intestinal immune responses observed from a simple intramuscular immunization offer great promise for a broad application of mRNA vaccination. Future research should focus on a comprehensive assessment of immune responses in the gut and lymph nodes focusing on eliciting tissue-resident memory T cells at these critical sites.[62]

In conclusion, mRNA-galsomes and mRNA-LNPs elicited comparable cellular immune responses to Gag in mice. However, mRNA-galsomes may offer an additional advantage by restoring iNKT cell polyfunctionality in PLWH, potentially leading to a more effective CD8$^+$ T cell response capable of suppressing viral replication. We hypothesize that both formulations are strong candidates for a therapeutic HIV vaccine, especially considering their capacity to enhance CD8$^+$ T cell functionality and the distinctive ability of galsomes to promote iNKT cell proliferation. Given the advantages of each platform, further investigation into a heterologous vaccine regimen incorporating both formulations could be highly beneficial in generating potent HIV-specific immune responses.

MATERIALS AND METHODS

Mice

Female CD45.1$^+$ C57BL/6NCrl mice, kindly provided by Prof. K. Movahedi (VUB, Brussels, Belgium), were housed at the breeding facility of the VUB, and were between 6 and 12 weeks of age at the start of the experiment. Female OT-I (ovalbumin-specific MHC class I-restricted CD8$^+$ T cells) mice carrying the transgenic T cell receptor for SIINFEKL, which is the most immunogenic epitope derived from ovalbumin presented in the context of MHC class I, were obtained from Charles River and bred under specific pathogen-free (SPF) conditions at the VUB. Female CD45.2$^+$ C57BL/6NCrl mice were purchased from Charles River and housed in SPF conditions in the VUB animal facility. Ethical approval from the ethical committee for animal experiments at the VUB was obtained for experiments with CD45.1 mice (21-275-7), C57BL/6NCrl mice (23-275-1) for the experiments with mRNA encoding Gag (21-275-4) and for organ collection of CD45.1 and OT-I mice (20-275-OC1).

Cell lines

The murine DC line DC2.4 was kindly provided by Prof. P. Midoux from the Centre National de Recherche Scientifique (CNRS) and Université d'Orléans. DC2.4 cells were cultured at 37°C with 5% CO_2 in Roswell Park Memorial Institute (RPMI) 1640 medium (VWR, Leuven, Belgium) supplemented with 5% fetal bovine serum (FBS) (TicoEurope, Amstelveen, the Netherlands), 50 μM β-mercaptoethanol (Sigma-Aldrich, Ghent, Belgium), and a mixture of supplements (SUP) consisting of 50 units/mL and 50 μg/mL penicillin and streptomycin (Sigma-Aldrich), respectively, 2 mM L-glutamine (Sigma-Aldrich), 1 mM sodium pyruvate (Sigma-Aldrich), and 100 μM non-essential amino acid solution (Lonza, Basel, Switzerland).

mRNA LNPs

Custom-made mRNA, fully substituted with 5-methylcytidine (m^5C) and ψ and capped with the Clean Cap AG system was obtained from Trilink Biotechnologies (San Diego, CA) for li80tOVA[63] and mRNA encoding the Gag HxB2 consensus gene.[64] The lipid components (C12-200, DSPC, cholesterol, PEG-DMG) for LNP formulation were dissolved in ethanol with a component molar ratio of ∼50:10:38.5:1.5 resulting in a final concentration of 10 mM total lipid. In the LNP formulation with α-GC, 0.02 mol % of the total lipid amount was replaced by α-GC (dissolved in DMSO). mRNA was dissolved in 25 mM sodium acetate buffer at pH 4.0 to obtain a final C12-200/mRNA weight ratio of 10:1. The organic and aqueous solutions were mixed using the Ignite NanoAssmblr (Precision NanoSystems) at a total flow rate of 12 mL/min and a flow rate ratio of 3:1 RNA/lipid. The resting suspension was dialyzed against a 1,000-fold volume PBS buffer at pH 7.4. For frozen storage at −70°C, the mRNA-LNP formulations were prepared with a C12-200/mRNA weight ratio of 20:1, 0.01 mol % α-GC and were dialyzed in Tris-buffer. The ionizable LNP formulations were subjected to a size and zeta potential quality control using a Malvern Zetasizer nano-ZS (Malvern Instruments, Worcestershire, UK). The Quant-iT RiboGreen RNA Assay was used to determine mRNA encapsulation and concentration in lipid particle formulations following the manufacturer's protocols (Thermo Fisher Scientific, Waltham, MA). To release and detect the encapsulated mRNA content, mRNA particles were diluted in TE buffer containing 1% (v/v) Triton X-100 (Sigma-Aldrich) and incubated for 10 min at 37°C, while the free (not-encapsulated) mRNA content was directly measured after particle dilution in TE buffer.

BMDCs

BMDCs were generated by culturing bone marrow from CD45.1$^+$ mice with 20 ng/mL GM-CSF (Immunotools, Friesoythe, Germany) for 6 days in RPMI, 5% SUP, 5% fetal clone I serum (FCI) (Thermo Fisher Scientific, Brussels, Belgium) and 50 μM β-mercaptoethanol (from here on referred to as RPMI+). To assess inflammasome

activation by nucleoside-modified mRNA-LNP or mRNA-galsomes, cells were incubated for 17 h with 4 μg/mL mRNA containing LNPs or galsomes and supernatant was stored at −20°C until analysis. Positive controls included 0.5 μg/mL LPS (kindly provided by Prof. Karine Breckpot, VUB) with or without 2 mM adenosine 5′-triphopshate (Merck, Darmstadt, Germany), and blocking of gasdermin D was performed with 50 μM NSA (Selleck Chemicals, TX). An ELISA for murine IL-1β (BioLegend, San Diego, CA) was performed according to the manufacturer's instructions.

In vivo adoptive transfer assay

Spleens were harvested from 6- to 10-week-old female OT-I mice and meshed through a 40 μM nylon filter (pluriSelect, Leipzig, Germany). After 2 min of incubation with red blood cell lysis buffer and washing with Dulbecco's phosphate-buffered saline (dPBS) (VWR), $CD8^+$ T cells were positively selected via magnet-activated cell sorting (MACS) according to the manufacturer's protocol. In brief, OT-I splenocytes were incubated in MACS buffer (0.5% BSA, 2 mM EDTA in PBS) with CD8a microbeads (Miltenyi, Bergisch Gladbach, Germany) for 10 min at 4°C and loaded onto an LD column. After flushing the column, $CD8a^+$ cells were resuspended in dPBS and stained with 1 μM CellTrace Violet (CTV) (Thermo Fisher Scientific) for 20 min in the dark at 37°C. Staining was neutralized with 5 times the volume RPMI+. Two days following the intravenous injection of 2×10^6 labeled cells, $CD45.1^+$ mice received intramuscular immunization with LNPs or galsomes containing 2 μg of modified (m^5C, ψ)-modified li80tOVA mRNA. After 3 days, spleens were collected and single-cell suspensions were prepared as described above. Inguinal and mesenteric lymph nodes were obtained and incubated with 0.1 U/mL Liberase TL (Roche, Basel, Switzerland) in Hank's balanced salt solution (HBSS) (Lonza) on ice. After 30 min at 37°C, lymph nodes were placed on ice, passed through a 70 μM nylon filter (pluriSelect), and rinsed in dPBS before staining for flow cytometry analysis to assess T cell proliferation, iNKT cells, as well as antigen presentation by DC subsets.

Identification of murine DCs and antigen presentation

C57BL/6NCrl mice were immunized intramuscularly with 2 μg of ψ/m^5C-modified li80tOVA mRNA encapsulated in LNPs or galsomes, with a total volume of 50 μL, or PBS as a negative control. One or 3 days post injection, inguinal and mesenteric lymph nodes, as well as the ileum, were isolated. Single suspensions of inguinal and mesenteric lymph nodes were prepared as previously described. For isolation of lamina propria cells, a protocol provided by Prof. Camilla Hartmann Friis Hansen and Prof. Axel Kornerup Hansen (University of Copenhagen) was followed.[65] In brief, the ileum was excised cranial to the ileocecal valve. After removing the Peyer's patches, longitudinal sections of ileum were cut into 0.5–1 cm pieces. The epithelial layer was disassociated and filtered from the underlying lamina propria by suspending the ileum sections in HBSS containing 5 mM EDTA (Thermo Fischer Scientific) and 5% FBS (Tico-Europe) for 20 min under vigorous shaking (150 rpm) at 37°C. Subsequently, to obtain the lamina propria leukocytes, the remaining tissue was digested using an enzyme mixture containing 0.04 mg/mL DNAse I (Sigma-Aldrich) and 1.5 mg/mL collagenase II (Sigma-Aldrich) in HBSS with 10% FBS and 0.75 mg/mL $CaCl_2$ (Sigma-Aldrich) and incubated for 40 min under rigorous shaking (250 rpm) at 37°C. Finally, leukocytes were purified using Lympholyte-M (Cedarlane Laboratories, Ontario, Canada) cell separation medium and spun down at 800 × *g* for 20 min. Following buffy coat isolation, leukocytes were washed twice in dPBS before staining for flow cytometry for antigen presentation by DC subsets.

Immunization and ELISPOT analysis

C57BL/6NCrl mice received two intramuscular immunizations with 0.5, 2, or 5 μg of ψ/m^5C Gag Hxb2 mRNA encapsulated LNPs or galsomes in a total volume of 50 μL, or PBS as a negative control with a 2-week interval between injections. Five days after the second injection, spleens were harvested and a single-cell suspension was prepared as described earlier. Subsequently, a murine IFN-γ ELISPOT (Diaclone, Medix Biochemica, Espoo, Finland) was conducted according to the manufacturer's instructions. In brief, 2.5×10^5 splenocytes or mesenteric lymph node single-cell suspensions were seeded on a capture antibody-coated Multiscreen-IP 0.45 μM plate (Millipore, Merck, Darmstadt, Germany) and stimulated with 5 μg/mL HIV subtype B (consensus) Gag-peptide pool (ARP-12425, NIH HIV reagent program) or 0.2% DMSO in RPMI+ as a negative control or CD3/CD28 Dynabeads (1/150) (Gibco, Thermo Fisher Scientific) as a positive control. After a 20-h incubation at 37°C and 5% CO_2, the plate was washed with 0.05% Tween 20-PBS and incubated with the detection antibody for 1.5 h at room temperature. Following this, the plate was washed, incubated with streptavidin-alkaline phosphatase and incubated for 1 h at room temperature. Subsequently, the plate underwent additional washing steps with Tween 20-PBS and distilled water followed by a 15-min incubation with the substrate. After a final washing step with distilled water, plates were air-dried overnight at 4°C and the readout was conducted the next day using the AID ELISPOT reader (Auto-immun Diagnostika, Strassberg, Germany). Any splenocytes not used in the assay were frozen in Cryostor (STEMCELL Technologies, Vancouver, Canada) and stored in liquid nitrogen. To identify the specific epitopes responsible for the reactivity toward Gag, ELISPOT assays were also performed with the Gag HIV subtype B (consensus) peptide array (ARP-8117, NIH HIV reagent program) on frozen splenocyte samples. This was confirmed on fresh samples, initially in pools of 10–12 overlapping peptides and subsequently in separate peptides at a concentration of 5 μg/mL.

Intracellular cytokine production from T cells

To assess cytokine production from T cells, spleen and lymph node single-cell suspensions were co-cultured with pulsed DC 2.4 cells at a ratio 10:1 (single-cell suspensions/DC2.4). Pulsing of DC2.4 cells was carried out using 0.5% DMSO in RPMI+ as a negative control or with 10 μg/mL of each of the selected Gag-peptides for 2 h at 37°C. As a positive control, spleen or lymph node single-cell suspensions were treated with 40 ng/mL phorbol-12-myristate-13-acetate (Merck) and 1 μg/mL ionomycin (Merck). After 4 h,

2 mM monensin and 5 μg/mL brefeldin A (BioLegend, San Diego, CA) were added to the cultures to stop cytokine secretion. The following day, cells were isolated and analyzed by flow cytometry.

In vivo cytotoxicity assay

C57BL/6NCrl mice were immunized as described above. Following this, splenocytes were isolated from naive C57BL/6NCrl mice and split into two equal portions. One part was pulsed with 10 μg/mL of each of the five selected Gag-peptides for 2 h at 37°C. Subsequently, the unpulsed cells were stained with 0.15 μM CTV and the pulsed cells were stained with 1.5 μM CTV. The two fractions were combined, and 10–20 million cells were injected intravenously into the immunized or PBS control mice 4 days after the booster injection. After 20 h, the spleen, and inguinal and mesenteric lymph nodes were harvested and single-cell suspensions were analyzed using flow cytometry. The percentage lysis was calculated based on the following formula:

$$\% \text{ lysis} = \frac{1-\frac{\text{CTV high immunized}}{\text{CTV low immunized}}}{\frac{\text{CTV high PBS control}}{\text{CTV low PBS control}}} \times 100$$

Flow cytometry

Fluorescently labeled antibodies (detailed description in Table S1) against CD11c, CD103, MHC class I bound to SIINFEKL (MHCI-S), CD86 and CD45.2 were purchased from BioLegend; CD45.1, CD3e, CD4, CD11b, CD8a, IFN-γ, TNF-α, and IL-2 were obtained from BD Biosciences (Erembodegem, Belgium) and MHC class II was purchased from eBiosience (Thermo Fisher Scientific). The mCD1d PBS-57 PE tetramer was obtained from the NIH Tetramer core facility. Cells were washed with dPBS and subsequently incubated for 20 min at room temperature in the dark with Fixable Viability Dye eFluor 450 or 780 (Thermo Fisher Scientific), diluted in dPBS. Afterwards, various combinations of antibodies diluted in FACS buffer (1% BSA, 0.1% sodium azide in dPBS) were added and incubated for 30 min at 4°C in the dark or 45 min for mCD1d PBS-57. IL-2, IFN-γ, and TNF-α production were assessed in $CD4^{+}$ or $CD8^{+}$ T cells via intracellular cytokine staining with the Cyto-Fast Fix/Perm buffer set (BioLegend), according to the manufacturer's instructions. In brief, cells were fixed for 20 min at room temperature with the Cyto-Fast Fix/Perm solution; subsequent washing steps were performed with Cyto-Fast Perm/Wash solution. After staining for 30 min at 4°C, samples were resuspended in FACS buffer. Samples were analyzed using the BD LSRFortessa or BD FACSymphony A1 Cell Analyzer (BD Bioscience, Franklin Lakes, NJ) at the VUB Flowcore.

Data analysis and statistics

Flow cytometry data were analyzed using FlowLogic 10.8.1 software (Inivai, Melbourne, Australia) and FlowJo v.9 software (Ashland, OR) at the VUB Flowcore. Statistical analysis was performed using the GraphPad Prism Software 9.3.1 (GraphPad Software, San Diego, CA). Data are represented as median ± interquartile range and a Kruskal-Wallis test with Dunn's multiple comparison test or Mann-Whitney U test was used for statistical comparison, unless otherwise indicated in the figure legend. p values of <0.05 were considered significant.

DATA AND CODE AVAILABILITY

All data generated during this study are included in the article and its supplemental information. The data that support the findings of this study are available from the corresponding author upon request.

ACKNOWLEDGMENTS

The authors wish to thank the NIH Tetramer Core Facility for providing the mCD1d PBS-57 tetramer. This work was supported by a grant from the Research Foundation Flanders (FWO-V, GOE8920N) to J.L.A. S.D., S.M., and I.A. are doctoral fellows from the FWO-V, with grant nos. 1S30319N, 1S73120N, and 1S40923N, respectively. R.V. is a postdoctoral fellow from FWO-V (grant no. 1275023N). I.L. would like to acknowledge funding from Ghent University Concerted Research Action (grant no. BOF21/GOA/033) and the FWO-V with grant no. G040319N. Furthermore, we would like to thank Prof. Dr. Guido Vanham for the critical evaluation of the results.

AUTHOR CONTRIBUTIONS

S.D., S.d.R., J.L.A., I.A., S.M., and R.V. designed the experiments. S.D., S.d.R., J.C., J.M., S.V., and T.L. contributed to the methodology. S.D. and S.d.R. collected and analyzed the data. S.D., S.d.R., and J.LA. wrote the manuscript. S.D., S.d.R., T.L., J.C., J.M., I.A., S.M., R.V., I.L., and J.L.A. reviewed the manuscript.

DECLARATION OF INTERESTS

R.V. and I.L. are contributors to patent applications no. WO2020058239A1 (Therapeutic nanoparticles and methods of use thereof) and no. WO2023209103 (Prevention and treatment of infections with intracellular bacteria), together with I.A.

DECLARATION OF GENERATIVE AI AND AI-ASSISTED TECHNOLOGIES IN THE WRITING PROCESS

During the preparation of this work the author(s) used ChatGPT and Copilot to improve language and readability. After using this tool/service, the author(s) reviewed and edited the content as needed and take(s) full responsibility for the content of the publication.

SUPPLEMENTAL INFORMATION

Supplemental information can be found online at https://doi.org/10.1016/j.omtn.2024.102372.

REFERENCES

1. Estes, J.D., Kityo, C., Ssali, F., Swainson, L., Makamdop, K.N., Del Prete, G.Q., Deeks, S.G., Luciw, P.A., Chipman, J.G., Beilman, G.J., et al. (2017). Defining total-body AIDS-virus burden with implications for curative strategies. Nat. Med. *23*, 1271–1276. https://doi.org/10.1038/nm.4411.
2. Deeks, S.G., Lewin, S.R., and Havlir, D.V. (2013). The End of AIDS: HIV Infection as a Chronic Disease. Lancet *382*, 1525–1533. https://doi.org/10.1016/S0140-6736(13)61809-7.
3. Paiardini, M., and Müller-Trutwin, M. (2013). HIV-associated chronic immune activation. Immunol. Rev. *254*, 78–101. https://doi.org/10.1111/IMR.12079.
4. Estes, J.D., Kityo, C., Ssali, F., Swainson, L., Makamdop, K.N., Del Prete, G.Q., Deeks, S.G., Luciw, P.A., Chipman, J.G., Beilman, G.J., et al. (2017). Defining total-body AIDS-virus burden with implications for curative strategies. Nat. Med. *23*, 1271–1276. https://doi.org/10.1038/nm.4411.
5. Allard, S.D., De Keersmaecker, B., de Goede, A.L., Verschuren, E.J., Koetsveld, J., Reedijk, M.L., Wylock, C., De Bel, A.V., Vandeloo, J., Pistoor, F., et al. (2012). A phase I/IIa immunotherapy trial of HIV-1-infected patients with Tat, Rev and Nef expressing dendritic cells followed by treatment interruption. Clin. Immunol. *142*, 252–268. https://doi.org/10.1016/j.clim.2011.10.010.
6. Leal, L., Guardo, A.C., Morón-López, S., Salgado, M., Mothe, B., Heirman, C., Pannus, P., Vanham, G., van den Ham, H.J., Gruters, R., et al. (2018). Phase I clinical trial of an intranodally administered mRNA-based therapeutic vaccine against HIV-1 infection. AIDS *32*, 2533–2545. https://doi.org/10.1097/QAD.0000000000002026.

7. Genovese, L., Nebuloni, M., and Alfano, M. (2013). Cell-mediated immunity in elite controllers naturally controlling hiv viral load. Front. Immunol. *4*, 86. https://doi.org/10.3389/fimmu.2013.00086.

8. Okulicz, J.F. (2012). Elite controllers and long-term nonprogressors: Models for HIV vaccine development? at OMICS International. J. Aids Clin. Res. *3*. https://doi.org/10.4172/2155-6113.S8-005.

9. Deeks, S.G., and Walker, B.D. (2007). Human Immunodeficiency Virus Controllers: Mechanisms of Durable Virus Control in the Absence of Antiretroviral Therapy. Immunity *27*, 406–416. https://doi.org/10.1016/j.immuni.2007.08.010.

10. Hersperger, A.R., Pereyra, F., Nason, M., Demers, K., Sheth, P., Shin, L.Y., Kovacs, C.M., Rodriguez, B., Sieg, S.F., Teixeira-Johnson, L., et al. (2010). Perforin Expression Directly Ex Vivo by HIV-Specific CD8+ T-Cells Is a Correlate of HIV Elite Control. PLoS Pathog. *6*, e1000917. https://doi.org/10.1371/JOURNAL.PPAT.1000917.

11. Betts, M.R., Nason, M.C., West, S.M., De Rosa, S.C., Migueles, S.A., Abraham, J., Lederman, M.M., Benito, J.M., Goepfert, P.A., Connors, M., et al. (2006). HIV non-progressors preferentially maintain highly functional HIV-specific CD8+ T cells. Blood *107*, 4781–4789. https://doi.org/10.1182/blood-2005-12-4818.

12. Migueles, S.A., Laborico, A.C., Shupert, W.L., Sabbaghian, M.S., Rabin, R., Hallahan, C.W., Van Baarle, D., Kostense, S., Miedema, F., McLaughlin, M., et al. (2002). HIV-specific CD8+ T cell proliferation is coupled to perforin expression and is maintained in nonprogressors. Nat. Immunol. *3*, 1061–1068. https://doi.org/10.1038/ni845.

13. Migueles, S.A., Osborne, C.M., Royce, C., Compton, A.A., Joshi, R.P., Weeks, K.A., Rood, J.E., Berkley, A.M., Sacha, J.B., Cogliano-Shutta, N.A., et al. (2008). Article Lytic Granule Loading of CD8 + T Cells Is Required for HIV-Infected Cell Elimination Associated with Immune Control. Immunity *29*, 1009–1021. https://doi.org/10.1016/j.immuni.2008.10.010.

14. Buggert, M., Nguyen, S., de Oca, G.S.M., Bengsch, B., Darko, S., Ransier, A., Roberts, E.R., del Alcazar, D., Brody, I.B., Vella, L.A., et al. (2018). Identification and characterization of HIV-specific resident memory CD8+ T cells in human lymphoid tissue. Sci. Immunol. *3*, eaar4526. https://doi.org/10.1126/SCIIMMUNOL.AAR4526.

15. Singh, D., Ghate, M., Godbole, S., Kulkarni, S., and Thakar, M. (2018). Functional invariant natural killer t cells secreting cytokines are associated with non-progressive human immunodeficiency virus-1 infection but not with suppressive anti-retroviral treatment. Front. Immunol. *9*, 372840. https://doi.org/10.3389/FIMMU.2018.01152/BIBTEX.

16. Kim, C.H., Johnston, B., and Butcher, E.C. (2002). Trafficking machinery of NKT cells: Shared and differential chemokine receptor expression among Vα24+Vβ11+ NKT cell subsets with distinct cytokine-producing capacity. Blood *100*, 11–16. https://doi.org/10.1182/blood-2001-12-0196.

17. Fleuridor, R., Wilson, B., Hou, R., Landay, A., Kessler, H., and Al-Harthi, L. (2003). CD1d-restricted natural killer T cells are potent targets for human immunodeficiency virus infection. Immunology *108*, 3–9. https://doi.org/10.1046/j.1365-2567.2003.01560.x.

18. van der Vliet, H.J.J., von Blomberg, B.M.E., Hazenberg, M.D., Nishi, N., Otto, S.A., van Benthem, B.H., Prins, M., Claessen, F.A., van den Eertwegh, A.J.M., Giaccone, G., et al. (2002). Selective Decrease in Circulating V 24+V 11+ NKT Cells During HIV Type 1 Infection. J. Immunol. *168*, 1490–1495. https://doi.org/10.4049/jimmunol.168.3.1490.

19. Paquin-Proulx, D., Lal, K.G., Phuang-Ngern, Y., Creegan, M., Tokarev, A., Suhkumvittaya, S., Alrubayyi, A., Kroon, E., Pinyakorn, S., Slike, B.M., et al. (2021). Preferential and persistent impact of acute HIV-1 infection on CD4 + iNKT cells in colonic mucosa. Proc. Natl. Acad. Sci. USA *118*, e2104721118. https://doi.org/10.1073/pnas.2104721118/-/DCSupplemental.

20. van der Vliet, H.J.J., van Vonderen, M.G.A., Molling, J.W., Bontkes, H.J., Reijm, M., Reiss, P., van Agtmael, M.A., Danner, S.A., van den Eertwegh, A.J.M., von Blomberg, B.M.E., and Scheper, R.J. (2006). Cutting Edge: Rapid Recovery of NKT Cells upon Institution of Highly Active Antiretroviral Therapy for HIV-1 Infection. J. Immunol. *177*, 5775–5778. https://doi.org/10.4049/jimmunol.177.9.5775.

21. Ibarrondo, F.J., Wilson, S.B., Hultin, L.E., Shih, R., Hausner, M.A., Hultin, P.M., Anton, P.A., Jamieson, B.D., and Yang, O.O. (2013). Preferential depletion of gut CD4-expressing iNKT cells contributes to systemic immune activation in HIV-1 infection. Mucosal Immunol. *6*, 591–600. https://doi.org/10.1038/MI.2012.101.

22. Moll, M., Kuylenstierna, C., Gonzalez, V.D., Andersson, S.K., Bosnjak, L., Sönnerborg, A., Quigley, M.F., and Sandberg, J.K. (2009). Severe functional impairment and elevated PD-1 expression in CD1d-restricted NKT cells retained during chronic HIV-1 infection. Eur. J. Immunol. *39*, 902–911. https://doi.org/10.1002/eji.200838780.

23. Laczko, D., Hogan, M.J., Toulmin, S.A., Hicks, P., Lederer, K., Gaudette, B.T., O, D.C., Amanat, F., Muramatsu, H., III, T.H.O., et al. (2020). A Single Immunization with Nucleoside-Modified mRNA Vaccines Elicits Strong Cellular and Humoral Immune Responses against SARS-CoV-2 in Mice. Immunity *53*, 19–21. Bizzarro. https://doi.org/10.1007/s00134-020-05991-x.

24. Arunachalam, P.S., Scott, M.K.D., Hagan, T., Li, C., Feng, Y., Wimmers, F., Grigoryan, L., Trisal, M., Edara, V.V., Lai, L., et al. (2021). Systems vaccinology of the BNT162b2 mRNA vaccine in humans. Nature *596*, 410–416. https://doi.org/10.1038/s41586-021-03791-x.

25. Baden, L.R., El Sahly, H.M., Essink, B., Kotloff, K., Frey, S., Novak, R., Diemert, D., Spector, S.A., Rouphael, N., Creech, C.B., et al. (2021). Efficacy and Safety of the mRNA-1273 SARS-CoV-2 Vaccine. N. Engl. J. Med. *384*, 403–416. https://doi.org/10.1056/nejmoa2035389.

26. Polack, F.P., Thomas, S.J., Kitchin, N., Absalon, J., Gurtman, A., Lockhart, S., Perez, J.L., Pérez Marc, G., Moreira, E.D., Zerbini, C., et al. (2020). Safety and Efficacy of the BNT162b2 mRNA Covid-19 Vaccine. N. Engl. J. Med. *383*, 2603–2615. https://doi.org/10.1056/nejmoa2034577.

27. Weber, J.S., Carlino, M.S., Khattak, A., Meniawy, T., Ansstas, G., Taylor, M.H., Kim, K.B., McKean, M., Long, G.V., Sullivan, R.J., et al. (2024). Individualised neoantigen therapy mRNA-4157 (V940) plus pembrolizumab versus pembrolizumab monotherapy in resected melanoma (KEYNOTE-942): a randomised, phase 2b study. Lancet *403*, 632–644. https://doi.org/10.1016/S0140-6736(23)02268-7.

28. Lorentzen, C.L., Haanen, J.B., Met, Ö., and Svane, I.M. (2022). Clinical advances and ongoing trials on mRNA vaccines for cancer treatment. Lancet Oncol. *23*, e450–e458. https://doi.org/10.1016/S1470-2045(22)00372-2.

29. Saunders, K.O., Pardi, N., Parks, R., Santra, S., Mu, Z., Sutherland, L., Scearce, R., Barr, M., Eaton, A., Hernandez, G., et al. (2021). Lipid nanoparticle encapsulated nucleoside-modified mRNA vaccines elicit polyfunctional HIV-1 antibodies comparable to proteins in nonhuman primates. npj Vaccines *6*, 50. https://doi.org/10.1038/s41541-021-00307-6.

30. Mu, Z., Wiehe, K., Saunders, K.O., Henderson, R., Cain, D.W., Parks, R., Martik, D., Mansouri, K., Edwards, R.J., Newman, A., et al. (2022). mRNA-encoded HIV-1 Env trimer ferritin nanoparticles induce monoclonal antibodies that neutralize heterologous HIV-1 isolates in mice. Cell Rep. *38*, 110514. https://doi.org/10.1016/j.celrep.2022.110514.

31. Leggat, D.J., Cohen, K.W., Willis, J.R., Fulp, W.J., DeCamp, A.C., Kalyuzhniy, O., Cottrell, C.A., Menis, S., Finak, G., Ballweber-Fleming, L., et al. (2022). Vaccination induces HIV broadly neutralizing antibody precursors in humans. Science *378*, eadd6502. https://doi.org/10.1126/science.add6502.

32. Kiepiela, P., Ngumbela, K., Thobakgale, C., Ramduth, D., Honeyborne, I., Moodley, E., Reddy, S., De Pierres, C., Mncube, Z., Mkhwanazi, N., et al. (2007). CD8 + T-cell responses to different HIV proteins have discordant associations with viral load. Nat. Med. *13*, 46–53. https://doi.org/10.1038/nm1520.

33. Moyo, N., Vogel, A.B., Buus, S., Erbar, S., Wee, E.G., Sahin, U., and Hanke, T. (2019). Efficient Induction of T Cells against Conserved HIV-1 Regions by Mosaic Vaccines Delivered as Self-Amplifying mRNA. Mol. Ther. Methods Clin. Dev. *12*, 32–46. https://doi.org/10.1016/j.omtm.2018.10.010.

34. Moyo, N., Wee, E.G., Korber, B., Bahl, K., Falcone, S., Himansu, S., Wong, A.L., Dey, A.K., Feinberg, M., and Hanke, T. (2020). Tetravalent immunogen assembled from conserved regions of HIV-1 and delivered as mRNA demonstrates potent preclinical T-cell immunogenicity and breadth. Vaccines *8*, 360. https://doi.org/10.3390/vaccines8030360.

35. Valentin, A., Bergamaschi, C., Rosati, M., Angel, M., Burns, R., Agarwal, M., Gergen, J., Petsch, B., Oostvogels, L., Loeliger, E., et al. (2022). Comparative immunogenicity of an mRNA/LNP and a DNA vaccine targeting HIV gag conserved elements in macaques. Front. Immunol. *13*, 945706. https://doi.org/10.3389/fimmu.2022.945706.

36. Verbeke, R., Lentacker, I., Breckpot, K., Janssens, J., Van Calenbergh, S., De Smedt, S.C., and Dewitte, H. (2019). Broadening the Message: A Nanovaccine Co-loaded with Messenger RNA and α-GalCer Induces Antitumor Immunity through

Conventional and Natural Killer T Cells. ACS Nano *13*, 1655–1669. https://doi.org/10.1021/acsnano.8b07660.

37. Fujii, S.I., Shimizu, K., Smith, C., Bonifaz, L., and Steinman, R.M. (2003). Activation of natural killer T cells by α-galactosylceramide rapidly induces the full maturation of dendritic cells in vivo and thereby acts as an adjuvant for combined CD4 and CD8 T cell immunity to a coadministered protein. J. Exp. Med. *198*, 267–279. https://doi.org/10.1084/jem.20030324.

38. Guevara, M.L., Jilesen, Z., Stojdl, D., and Persano, S. (2019). Codelivery of mRNA with α-galactosylceramide using a new lipopolyplex formulation induces a strong antitumor response upon intravenous administration. ACS Omega *4*, 13015–13026. https://doi.org/10.1021/acsomega.9b00489.

39. Meulewaeter, S., Aernout, I., Deprez, J., Engelen, Y., De Velder, M., Franceschini, L., Breckpot, K., Van Calenbergh, S., Asselman, C., Boucher, K., et al. (2024). Alpha-galactosylceramide improves the potency of mRNA LNP vaccines against cancer and intracellular bacteria. J. Control. Release *370*, 379–391. https://doi.org/10.1016/j.jconrel.2024.04.052.

40. Tahtinen, S., Tong, A.J., Himmels, P., Oh, J., Paler-Martinez, A., Kim, L., Wichner, S., Oei, Y., McCarron, M.J., Freund, E.C., et al. (2022). IL-1 and IL-1ra are key regulators of the inflammatory response to RNA vaccines. Nat. Immunol. *23*, 532–542. https://doi.org/10.1038/s41590-022-01160-y.

41. Buggert, M., Nguyen, S., Oca, G.S.-M. de, Bengsch, B., Darko, S., Ransier, A., Roberts, E.R., del Alcazar, D., Brody, I.B., Vella, L.A., et al. (2018). Identification and characterization of HIV-specific resident memory CD8+ T cells in human lymphoid tissue. Sci. Immunol. *24*, 1–14. https://doi.org/10.3188/szf.2008.0435.

42. Ferre, A.L., Hunt, P.W., Critchfield, J.W., Young, D.H., Morris, M.M., Garcia, J.C., Pollard, R.B., Yee, H.F., Martin, J.N., Deeks, S.G., and Shacklett, B.L. (2009). Mucosal immune responses to HIV-1 in elite controllers : a potential correlate of immune control. Blood *113*, 3978–3989. https://doi.org/10.1182/blood-2008-10-182709.

43. Mörbe, U.M., Jørgensen, P.B., Fenton, T.M., von Burg, N., Riis, L.B., Spencer, J., and Agace, W.W. (2021). Human gut-associated lymphoid tissues (GALT); diversity, structure, and function. Mucosal Immunol. *14*, 793–802. https://doi.org/10.1038/s41385-021-00389-4.

44. Saez-Cirion, A., Lacabaratz, C., Lambotte, O., Versmisse, P., Urrutia, A., Boufassa, F., Barre-Sinoussi, F., Delfraissy, J.-F., Sinet, M., Pancino, G., et al. (2007). HIV controllers exhibit potent CD8 T cell capacity to suppress HIV infection ex vivo and peculiar cytotoxic T lymphocyte activation phenotype. Proc. Natl. Acad. Sci. *104*, 6776–6781. https://doi.org/10.1073/pnas.0611244104.

45. Ndhlovu, Z.M., Chibnik, L.B., Proudfoot, J., Vine, S., Mcmullen, A., Cesa, K., Porichis, F., Jones, R.B., Alvino, D.M., Hart, M.G., et al. (2013). High-dimensional immunomonitoring models of HIV-1 – specific CD8 T-cell responses accurately identify subjects achieving spontaneous viral control. Blood *121*, 801–811. https://doi.org/10.1182/blood-2012-06-436295.

46. Lenart, K., Hellgren, F., Ols, S., Yan, X., Cagigi, A., Cerveira, R.A., Winge, I., Hanczak, J., Mueller, S.O., Jasny, E., et al. (2022). A third dose of the unmodified COVID-19 mRNA vaccine CVnCoV enhances quality and quantity of immune responses. Mol. Ther. Methods Clin. Dev. *27*, 309–323. https://doi.org/10.1016/j.omtm.2022.10.001.

47. Gottschalk, C., Mettke, E., and Kurts, C. (2015). The role of invariant natural killer T cells in dendritic cell licensing, cross-priming, and memory CD8+ T cell generation. Front. Immunol. *6*, 379. https://doi.org/10.3389/fimmu.2015.00379.

48. Semmling, V., Lukacs-Kornek, V., Thaiss, C.A., Quast, T., Hochheiser, K., Panzer, U., Rossjohn, J., Perlmutter, P., Cao, J., Godfrey, D.I., et al. (2010). Alternative cross-priming through CCL17-CCR4-mediated attraction of CTLs toward NKT cell-licensed DCs. Nat. Immunol. *11*, 313–320. https://doi.org/10.1038/ni.1848.

49. Li, C., Lee, A., Grigoryan, L., Arunachalam, P.S., Scott, M.K.D., Trisal, M., Wimmers, F., Sanyal, M., Weidenbacher, P.A., Feng, Y., et al. (2022). Mechanisms of innate and adaptive immunity to the Pfizer-BioNTech BNT162b2 vaccine. Nat. Immunol. *23*, 543–555. https://doi.org/10.1038/s41590-022-01163-9.

50. Persson, E.K., Scott, C.L., Mowat, A.M., and Agace, W.W. (2013). Dendritic cell subsets in the intestinal lamina propria: Ontogeny and function. Eur. J. Immunol. *43*, 3098–3107. https://doi.org/10.1002/eji.201343740.

51. Baker, K., Qiao, S.W., Kuo, T.T., Aveson, V.G., Platzer, B., Andersen, J.T., Sandlie, I., Chen, Z., De Haar, C., Lencer, W.I., et al. (2011). Neonatal Fc receptor for IgG (FcRn) regulates cross-presentation of IgG immune complexes by CD8-CD11b+ dendritic cells. Proc. Natl. Acad. Sci. USA *108*, 9927–9932. https://doi.org/10.1073/PNAS.1019037108/-/DCSUPPLEMENTAL.

52. Wilson, N.S., Yang, B., Morelli, A.B., Koernig, S., Yang, A., Loeser, S., Airey, D., Provan, L., Hass, P., Braley, H., et al. (2012). ISCOMATRIX vaccines mediate CD8+ T-cell cross-priming by a MyD88-dependent signaling pathway. Immunol. Cell Biol. *90*, 540–552. https://doi.org/10.1038/ICB.2011.71.

53. Murray, M.P., Crosby, C.M., Marcovecchio, P., Hartmann, N., Chandra, S., Zhao, M., Khurana, A., Zahner, S.P., Clausen, B.E., Coleman, F.T., et al. (2022). Stimulation of a subset of natural killer T cells by CD103+ DC is required for GM-CSF and protection from pneumococcal infection. Cell Rep. *38*, 110209. https://doi.org/10.1016/j.celrep.2021.110209.

54. Ndeupen, S., Qin, Z., Jacobsen, S., Bouteau, A., Estanbouli, H., and Igyártó, B.Z. (2021). The mRNA-LNP platform's lipid nanoparticle component used in preclinical vaccine studies is highly inflammatory. iScience *24*, 103479. https://doi.org/10.1016/J.ISCI.2021.103479.

55. Guardo, A.C., Joe, P.T., Miralles, L., Bargalló, M.E., Mothe, B., Krasniqi, A., Heirman, C., García, F., Thielemans, K., Brander, C., et al. (2017). Preclinical evaluation of an mRNA HIV vaccine combining rationally selected antigenic sequences and adjuvant signals (HTI-TriMix). AIDS *31*, 321–332. https://doi.org/10.1097/QAD.0000000000001276.

56. Pollard, C., Rejman, J., De Haes, W., Verrier, B., Van Gulck, E., Naessens, T., De Smedt, S., Bogaert, P., Grooten, J., Vanham, G., and De Koker, S. (2013). Type I IFN counteracts the induction of antigen-specific immune responses by lipid-based delivery of mRNA vaccines. Mol. Ther. *21*, 251–259. https://doi.org/10.1038/mt.2012.202.

57. Frahm, N., Linde, C., and Brander, C. (2006). Identification of HIV-Derived, HLA Class I Restricted CTL Epitopes: Insights into TCR Repertoire, CTL Escape and Viral Fitness. HIV Mol. Immunol. *chapter I-A*, 3–28.

58. Herath, S., Le Heron, A., Colloca, S., Bergin, P., Patterson, S., Weber, J., Tatoud, R., and Dickson, G. (2015). Analysis of T cell responses to chimpanzee adenovirus vectors encoding HIV gag-pol-nef antigen. Vaccine *33*, 7283–7289. https://doi.org/10.1016/j.vaccine.2015.10.111.

59. Pardi, N., Hogan, M.J., Naradikian, M.S., Parkhouse, K., Cain, D.W., Jones, L., Moody, M.A., Verkerke, H.P., Myles, A., Willis, E., et al. (2018). Nucleoside-modified mRNA vaccines induce potent T follicular helper and germinal center B cell responses. J. Exp. Med. *215*, 1571–1588. https://doi.org/10.1084/jem.20171450.

60. Ishii, H., Terahara, K., Nomura, T., Takeda, A., Okazaki, M., Yamamoto, H., Tokusumi, T., Shu, T., and Matanoa, T. (2020). A Novel Immunogen Selectively Eliciting CD8þ T Cells but Not CD4+ T Cells Targeting Immunodeficiency Virus Antigens. J. Virol. *94*, 1–15.

61. Jawalagatti, V., and Kirthika, P. (2022). Oral mRNA Vaccines Against Infectious Diseases- A Bacterial Perspective. Front. Immunol. *13*, 1–8. https://doi.org/10.3389/fimmu.2022.884862.

62. Ganley, M., Holz, L.E., Minnell, J.J., de Menezes, M.N., Burn, O.K., Poa, K.C.Y., Draper, S.L., English, K., Chan, S.T.S., Anderson, R.J., et al. (2023). mRNA vaccine against malaria tailored for liver-resident memory T cells. Nat. Immunol. *24*, 1487–1498. https://doi.org/10.1038/s41590-023-01562-6.

63. Van Meirvenne, S., Straetman, L., Heirman, C., Dullaers, M., De Greef, C., Van Tendeloo, V., and Thielemans, K. (2002). Efficient genetic modification of murine dendritic cells by electroporation with mRNA. Cancer Gene Ther. *9*, 787–797. https://doi.org/10.1038/sj.cgt.7700499.

64. Van Gulck, E.R., Vanham, G., Heyndrickx, L., Coppens, S., Vereecken, K., Atkinson, D., Florence, E., Kint, I., Berneman, Z.N., and Van Tendeloo, V. (2008). Efficient in vitro expansion of human immunodeficiency virus (HIV)-specific T-cell responses by gag mRNA-electroporated dendritic cells from treated and untreated HIV type 1-infected individuals. J. Virol. *82*, 3561–3573. https://doi.org/10.1128/JVI.02080-07.

65. Zachariassen, L.F., Hansen, A.K., Krych, L., Nielsen, D.S., Holm, T.L., Tougaard, P., and Hansen, C.H.F. (2019). Cesarean section increases sensitivity to oxazolone-induced colitis in C57BL/6 mice. Mucosal Immunol. *12*, 1348–1357. https://doi.org/10.1038/S41385-019-0207-8.

OMTN, Volume *35*

Supplemental information

The role of mRNA-galsomes and LNPs in enhancing HIV-specific T cell responses across various lymphoid organs

Sigrid D'haese, Sabine den Roover, Rein Verbeke, Ilke Aernout, Sofie Meulewater, Joëlle Cosyns, Jessy Meert, Sarah Vanbellingen, Thessa Laeremans, Ine Lentacker, and Joeri L. Aerts

Supplemental material

Table S1: Antibodies used for flow cytometry analysis

Target	Fluorophore	Clone	Supplier
CD11c	PE-Cy-7a	N418	Biolegend
CD103	AF-700	2E7	
MHC-IS	APC	25-D1.16	
CD86	PE CF594	GL-1	
CD45.2	BV421	104	
	Spark NIR TM 685	104	
CD45.1	PE	A20	BD
CD3e	FITC	145-2C11	
CD4	PerCP-Cy5.5	RM4-5	
CD11b	PerCP-Cy5.5	M1/70	
CD8a	BV510	53-6.7	
IFN-γ	AF-700	XMG1.2	
TNF-α	PeCy7	MP6-XT22	
IL-2	APC	JES6-5H4	
MHC-II	PE	NIM-R4	eBioscience

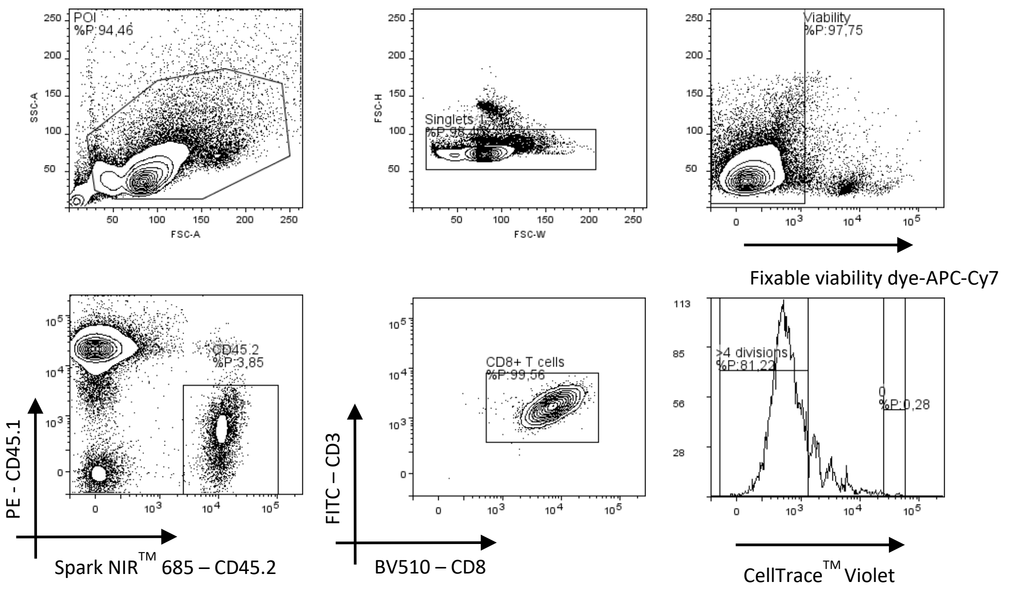

Figure S1: Gating strategy for the in vivo adoptive transfer assay. Viable cells were selected based on fixable viability dye and subsequently gated on $CD45.2^+$ to exclude host lymphocytes. Next, the transferred $CD8^+$ T cells were gated based on CD3 and CD8. Finally, proliferated cells were selected on dilution peaks, the final peak being the cells that divided more than four times.

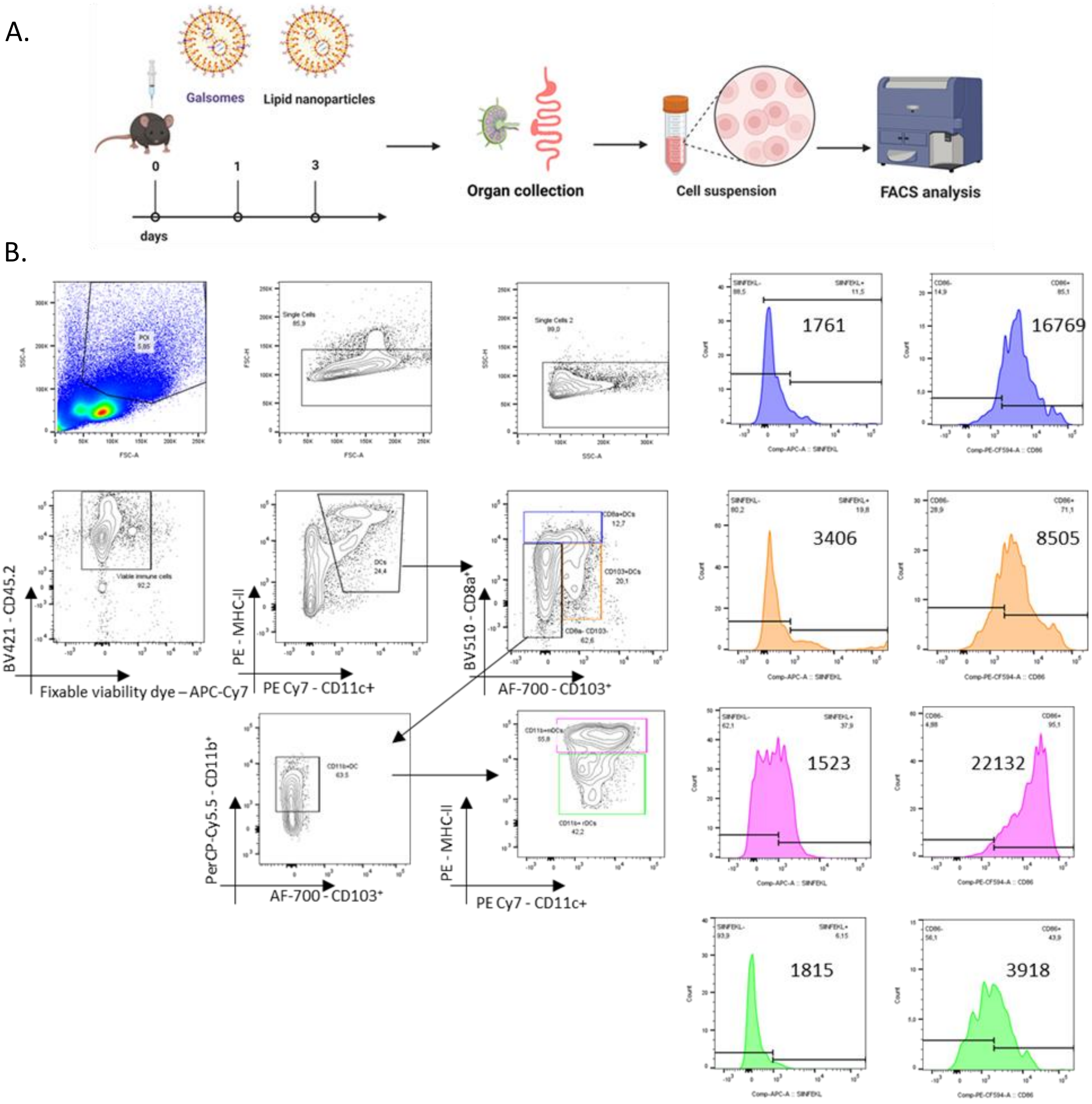

Figure S2. Experimental setup (A) and gating strategy antigen presentation and maturation in DCs in the ipsilateral lymph nodes (B). Gating was performed on the viable CD45.2^{+} cells. DCs were gated based on MHC II and CD11c double positivity. Subsequently, CD8a^{+} rDCs and CD103^{+} mDCs were identified and based on the double negative population, the CD11b^{+} m/r DCs were characterized. Within these subsets, presentation of SIINFEKL in MHC I and CD86 was assessed.

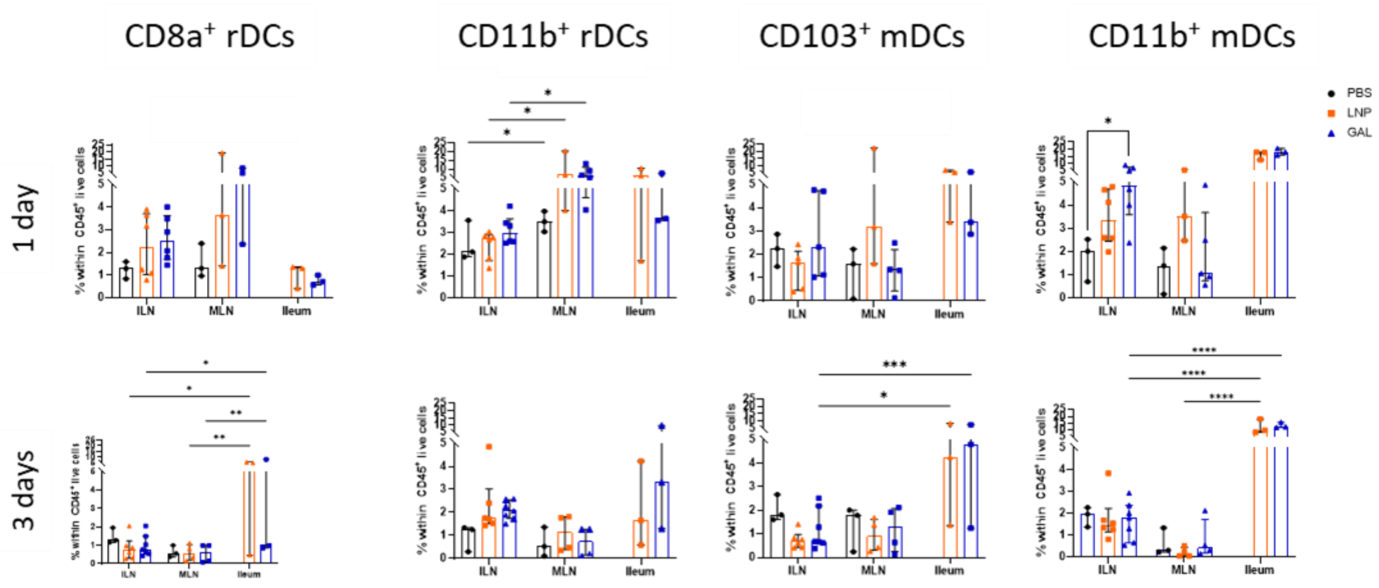

Figure S3. The identification of distinct DC subsets upon immunization with mRNA-LNP and mRNA-galsomes. The dynamics of various DC subsets in the inguinal (ILN) and mesenteric lymph nodes (MLN) and ileum following immunization with 2 µg Ψ/m^{5}C modified li80tOVA mRNA-LNP (LNP) and mRNA-galsomes (GAL). For ILN and MLN: each dot represents data from one mouse. For ileum: each dot represents data pooled from 2 mice. For the 24 and 72h time points, n=3 for PBS, n=6 for galsomes and n= 6. LNP. Median ± IQR. Mann-Whitney U-test, * p <0.05, ** p<0.01.

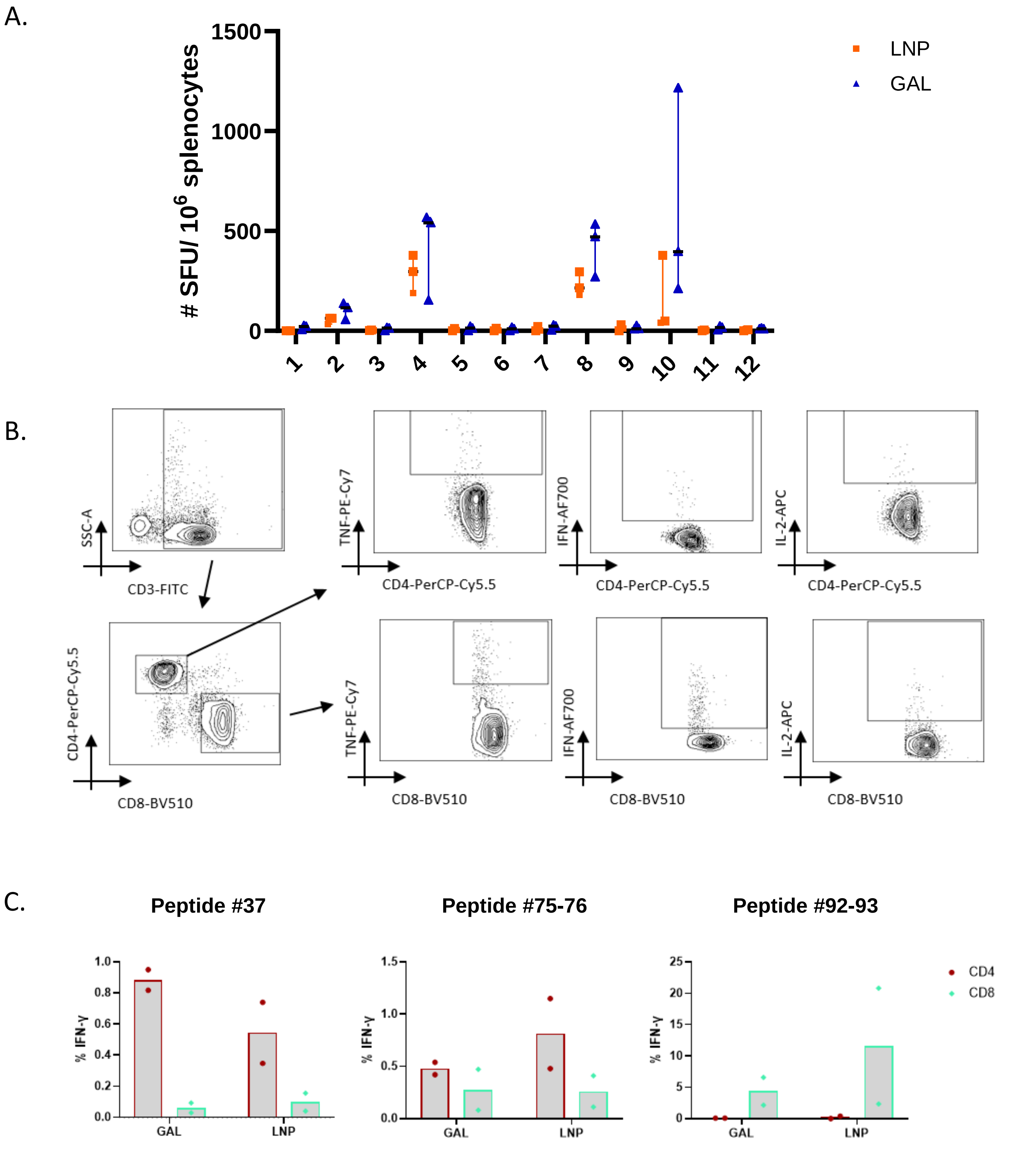

Figure S4. Identification of minimal H2k^b stimulatory epitopes within HIV gag. A) ELISPOT for IFN-γ was performed for 12 separate gag-peptide pools each containing 10-12 overlapping peptides. B) Gating strategy intracellular cytokine staining. Splenocytes were gated based on CD3 and subsequently CD4 and CD8. Next, we looked at TNF, IFN and IL-2 production within the CD4 and CD8 population. C) IFN-γ secretion was assessed in splenocytes from immunized mice in CD4$^+$ and CD8$^+$ T cell separately. In brief, cells were thawed and peptides were added before overnight stimulation in the presence of monensin and brefeldin. n=2, each dot represents data from one mouse, bar represents mean.

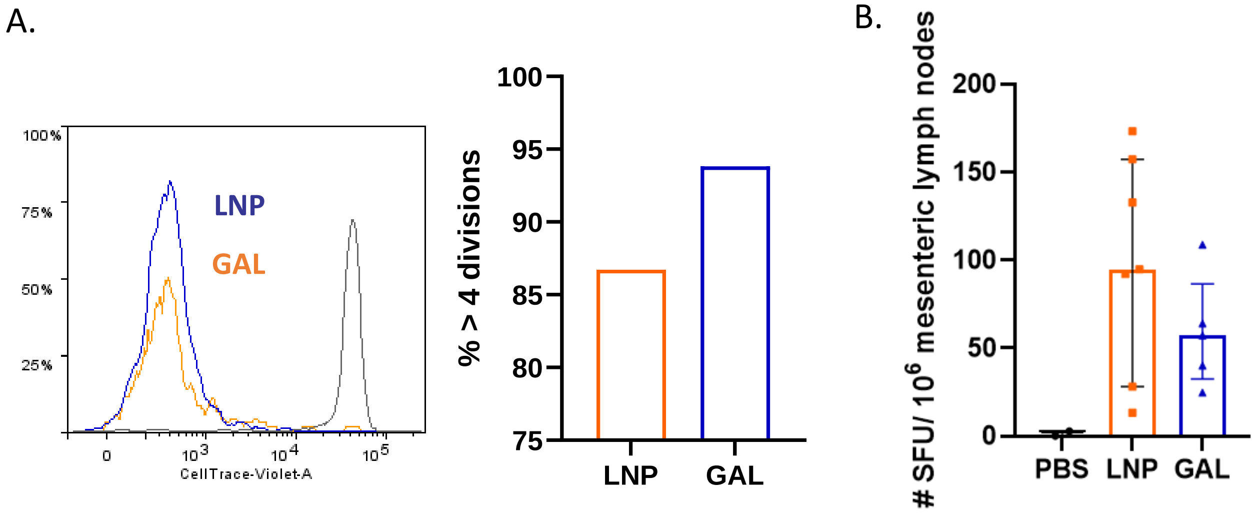

Figure S5. Immune response in the mesenteric lymph nodes. (A) CD8$^+$ T cells from OT-I mice were transferred to naive CD45.1$^+$ mice. After 2 days, naive CD45.1$^+$ mice were immunized with lipid nanoparticles (LNP) or galsomes (GAL) containing 2 µg ψ/m^{5}C-modified mRNA encoding li80tOVA. After 3 days, mice were sacrificed and proliferation of transferred CD8$^+$ T cells was assessed by flow cytometry in the mesenteric lymph nodes for one mouse in the GAL (blue) and LNP (orange) group. The PBS control is shown for the inguinal lymph nodes (gray). (B) Prime-boost immunization was performed with 2 µg Ψ/m^{5}C Gag mRNA containing galsomes and LNPs. Five days after boost injection, mesenteric lymph nodes were collected and used for IFN-γ ELISPOT analysis. Each symbol represents one mouse, n=5 for galsomes, n=7 for LNPs, n=2 for PBS controls. Median ± IQR, one outlier was removed in the GAL group.
